# Comparative Plastome Analysis of Three Amaryllidaceae Subfamilies: Insights into Variation of Genome Characteristics, Phylogeny, and Adaptive Evolution

**DOI:** 10.1155/2022/3909596

**Published:** 2022-03-24

**Authors:** Rui-Yu Cheng, Deng-Feng Xie, Xiang-Yi Zhang, Xiao Fu, Xing-Jin He, Song-Dong Zhou

**Affiliations:** Key Laboratory of Bio-Resources and Eco-Environment of Ministry of Education, College of Life Sciences, Sichuan University, 610065 Chengdu, Sichuan, China

## Abstract

In the latest APG IV classification system, Amaryllidaceae is placed under the order of Asparagus and includes three subfamilies: Agapanthoideae, Allioideae, and Amaryllidoideae, which include many economically important crops. With the development of molecular phylogeny, research on the phylogenetic relationship of Amaryllidaceae has become more convenient. However, the current comparative analysis of Amaryllidaceae at the whole chloroplast genome level is still lacking. In this study, we sequenced 18 Allioideae plastomes and combined them with publicly available data (a total of 41 plastomes), including 21 Allioideae species, 1 Agapanthoideae species, 14 Amaryllidoideae species, and 5 Asparagaceae species. Comparative analyses were performed including basic characteristics of genome structure, codon usage, repeat elements, IR boundary, and genome divergence. Phylogenetic relationships were detected using single-copy genes (SCGs) and ribosomal internal transcribed spacer sequences (ITS), and the branch-site model was also employed to conduct the positive selection analysis. The results indicated that all Amaryllidaceae species showed a highly conserved typical tetrad structure. The GC content and five codon usage indexes in Allioideae species were lower than those in the other two subfamilies. Comparison analysis of Bayesian and ML phylogeny based on SCGs strongly supports the monophyly of three subfamilies and the sisterhood among them. Besides, positively selected genes (PSGs) were detected in each of the three subfamilies. Almost all genes with significant posterior probabilities for codon sites were associated with self-replication and photosynthesis. Our study investigated the three subfamilies of Amaryllidaceae at the whole chloroplast genome level and suggested the key role of selective pressure in the adaptation and evolution of Amaryllidaceae.

## 1. Introduction

Amaryllidaceae belong to Asparagales and is a worldwide distributed family of monocotyledons [1]. Early APG II (Angiosperm Phylogeny Group II) classification believed that Amaryllidaceae could be merged with the genera *Allium* and *Agapanthus* based on phylogeny, or it could be divided into a single division [2]. According to the principle of merging small families, the latest revised version of APG IV [1] exhibited major changes, which divided Amaryllidaceae into three subfamilies: Allioideae (e.g., *Allium* spp.), Agapanthoideae (e.g., American bluebells), and Amaryllidoideae (e.g., daffodils and amaryllises). Meanwhile, the phylogenetic relationships among the three subfamilies have been extensively investigated [3–13], and three sister lineages were supported, often presenting Amaryllidoideae and Allioideae as sister lineages, with Agapanthoideae as sister to both.

Currently, more than 1,800 species have been recorded in Amaryllidaceae [14]; among them, the subfamily Allioideae occupies 13 genera and more than 900 species [15], which are widely distributed in the Northern Hemisphere and include many economically important crops, such as garlic, leek, onion, and shallot [16, 17]. The subfamily Amaryllidoideae also has approximately 900 species, which include many famous ornamental plants, such as *Crinum asiaticum*, *Clivia miniata*, and *Hippeastrum rutilum* [10, 18]. Agapanthoideae is a small subfamily of Amaryllidaceae, and only approximately 10 species have been reported, which are also famous ornamental cultivars and are widely cultivated worldwide. For the significant edible, medicinal, and ornamental values of species in Amaryllidaceae, research on these species has never stopped, which also provides valuable information for us to perform further research.

Beyond the phylogenetic studies conducted on the three subfamilies of Amaryllidaceae, genome and transcriptome data were also used to perform evolutionary and adaptive analyses on Amaryllidaceae species in recent years [11–13, 19–21]. Complete plastome sequences, which have a highly conserved genome structure and gene content and a low substitution rate, offer effective approaches for investigating the phylogeny, species divergence, and adaptive evolution of plant species [12, 22–26]. In particular, the substitution rates of the plastome are several times lower in the inverted repeat (IR) than SSC (small single-copy) regions [11, 23, 27–29]. We found that species from Allioideae exhibit lower GC content than relatives and lost some genes (e.g., *rps2*). Further studies suggested that 27 genes of Amaryllidaceae species possess positively selected sites (e.g., *matK*, *petD*, and *rbcL*), and 10 of them are owned by Allioideae species [12]. Of course, some Amaryllidoideae and Agapanthoideae plastome sequences have been released [30, 31]. However, most of the public chloroplast genomes are annotated with different methods, which will result in more or less annotation errors, and most previous studies have focused on Allioideae. No studies have investigated the difference in plastome structure and adaptive evolution among the three subfamilies.

In this study, a total of 36 chloroplast genomes were collected and reannotated using a uniform approach, including 21 Allioideae species (18 of which were sequenced and assembled here), one Agapanthoideae species, and 14 Amaryllidoideae species. Comparative plastome analyses were performed, and our objectives were to (1) gain insights into the plastome structure features of Amaryllidaceae; (2) investigate the genome variation among the three subfamilies; (3) reconstruct the phylogenetic relationships of Amaryllidaceae species; and (4) explore adaptive evolution based on selective analysis. Our studies will contribute to a comprehensive understanding of plastome evolution in Amaryllidaceae.

## 2. Materials and Methods

### 2.1. Taxon Sampling

In this study, we collected 41 plastid genomes representing three subfamilies of Amaryllidaceae and an outgroup of Asparagaceae. Among them, there were 21 Allioideae species, 1 Agapanthoideae species, 14 Amaryllidoideae species, and 5 Asparagaceae species. (GenBank accessions: Supplementary Table 2). Among all 41 plastomes, we assembled 18 plastomes, and fresh leaves were collected from the wild and then desiccated and stored in silica gel (Supplementary Table S1). Total genomic DNA was extracted from silica-dried leaves with a modified CTAB method with the default parameters [32]. Voucher specimens were deposited in the Sichuan University Herbarium (SZ). In addition, we downloaded 38 ITS sequences of Amaryllidaceae and Asparagaceae species from GenBank (GenBank accessions: Supplementary Table 3).

### 2.2. Plastome Genome Sequencing, Assembling, and Annotation

Total genomic DNA was sent to Novogene Technologies, Inc. (Beijing, China) for genome library construction and sequencing. The sequencing library was generated using the NEB Next® Ultra™ DNA Library Prep Kit for Illumina (NEB, United States) according to the manufacturer's recommendations, and index codes were added to each sample. Sequencing was executed using an Illumina NovaSeq 2500 sequencer (Illumina, San Diego, CA, United States). Then, the plastomes were de novo assembled by NOVOPlasty v2.7.1 [33] with clean data. To minimize the impact of distant starting seed sequences on the plastomes, we used a consistent seed sequence (A. cepa, GenBank No. KF769495) within species as a reference sequence. The bases or sequences that could not be confirmed were modified by designing primers for PCR amplification and performing first-generation sequencing. Gene annotations and IR region searches were undertaken using PGA software [34]. Three chloroplast genomes (*A. cepa*, *A. sativum*, and *A. chinense*) were set as reference sequences, and the results were adjusted manually in GENEIOUS R11 [35] based on comparisons with homologous genes of other species' plastomes. Circular plastome maps were drawn using the online program OGDRAW [36].

### 2.3. Sequence Basic Information and Sequence Divergence

Basic information statistics for all chloroplast sequences were performed using GENEIOUS R11, including the length and GC content of the genome sequences and the number of CDSs and genes in each category. Based on *A. listera* as a reference, mVISTA [37] was used to construct and visualize the whole-genome alignment of 36 plastomes.

### 2.4. Contraction and Expansion of IRs and Repeat Element Analysis

The program IRscope (https://irscope.shinyapps.io/irapp/) [38] was used to compare the boundaries between the IR and SC regions of the 36 species and then correct them manually. The Perl script MISA [39] was used to count the plastid SSRs, and the repetition thresholds were set as follows: mononucleotides 10 repeats, dinucleotides 5 repeats, trinucleotide 4 repeats, and tetranucleotides, pentanucleotides, and hexanucleotides have 3 repeats. We used the online REPuter program [40] to identify repeat sequences, including forward repeats, palindromic repeats, reverse repeats, and complementary repeats. The parameters were set as follows: (1) screen repeats with the sizes longer than 30 bp; (2) the sequence identity between two repeated sequences exceeding 90%; and (3) hamming distance = 3. All overlapping repeat sequences in the test results were removed.

### 2.5. Indices of Codon Usage

The protein-coding genes from the 36 plastomes were extracted, and all overlapping genes were removed for codon analysis. The final dataset included 65 consensus protein-coding genes for each species. Six values were used to estimate the degree of codon preference: relative synonymous codon usage (RSCU), codon adaptation index (CAI), codon bias index (CBI), effective number of codons (ENC), GC content of synonymous third codon positions (GC3s), and frequency of optimal codons (Fop) [41]. All the above values were calculated by the CodonW v1.4.2 program [42], and the heat map of all RSCUs was drawn using TBtools [43].

### 2.6. Phylogenetic Analyses

We reconstructed the phylogenetic relationships of Amaryllidaceae species based on the two datasets (including a 41-taxon plastome dataset and a separate dataset comprising 38 nuclear ITS sequences). For plastomes, all shared single-copy genes (SCGs) were extracted from the 41 taxa and then aligned using MAFFT program [44]. We adjusted all alignments manually using the GENEIOUS R11 software [34] and concatenated all of them into plastid supermatrices using PhyloSuite software [45]. For ITS, we aligned them using the MAFFT program [44] and then adjusted manually using GENEIOUS R11 [34]. Maximum likelihood analyses (ML) of the two datasets were performed using the RAxML v7.2.8 [46] under the GTRGAMMA model and 1000 bootstrap replicates. Bayesian inference (BI) was performed on the two datasets using the software MrBayes v3.2.7 [47] with the GTR+G substitution model. The Markov chain Monte Carlo (MCMC) algorithm was run for 2∗107 generations, and one tree was sampled every 1000 generations. The convergence of MCMC was determined by calculating the average standard deviation of split frequencies, and stationarity was considered to be reached when it fell below 0.01 and ESS > 200. We discarded the first 25% percent of the trees as burn-in and used the remaining trees to generate the 50% majority-rule consensus tree.

### 2.7. Positive Selected Pressure Analyses

The single-copy CDSs of all 36 species were extracted and further aligned using MUSCLE v3.6 software [48]. The DNA codon sequence alignments were further trimmed by TRIMAL v1.2 [49], and the final processing alignments were used for the positive selection analyses. The optimized branch-site model and Bayesian empirical Bayes (BEB) methods [50–52] were used to perform the related analysis. To identify genes under positive selection among the three subfamilies, the species of each subfamily was set as the foreground branch and compared with the other two subfamilies through the optimized branch-site model. The ratio (*ω*) of the nonsynonymous substitution rate to the synonymous substitution rate (Ka/Ks) was calculated using the PAML v4.8 package with the branch-site model [51]. The likelihood ratio test (LRT) was used to confirm the quality of the different sets above [53]. The Bayesian Empirical Bayes (BEB) method was used to statistically identify whether the selected sites were under positive selection (posterior probabilities ≥ 95%). We classified these genes as follows: *ω* < 1, *ω* = 1, and *ω* > 1 suggesting negative selection, neutral selection, and positive selection, respectively [54]. The gene that was positively selected and with a test *p* value < 0.05 was considered a positively selected gene (PSG) [40].

### 2.8. Ancestral Character-State Reconstructions

We conducted reconstructions of two vegetative features, namely, (i) bulb shape and (ii) leaf shape. All morphological feature information comes from field observations, specimen studies, or literature information [55–60]. The details of the above two characters are provided in Supplementary Table 11. The RASP v4 software [61] was used to reconstruct the ancestral traits of the leaf and bulb types. Amaryllidaceae bulbs were divided into three types, namely, (i) spherical, (ii) cylindrical, and (iii) ovoid, coded as A, B, and C, respectively. And the leaves were divided into six types, namely, (i) ribbon, (ii) wide bar, (iii)wide line, (iv) oval, (v) bar, and (vi) lanceolate, coded as a-f, respectively (Supplementary Table 11). The MCMC iterations were set to 100 million and sampled every 10,000 iterations. The first 50,000 iterations were set into burn-in.

## 3. Results

### 3.1. Chloroplast Features of Species

**Figure 1:**
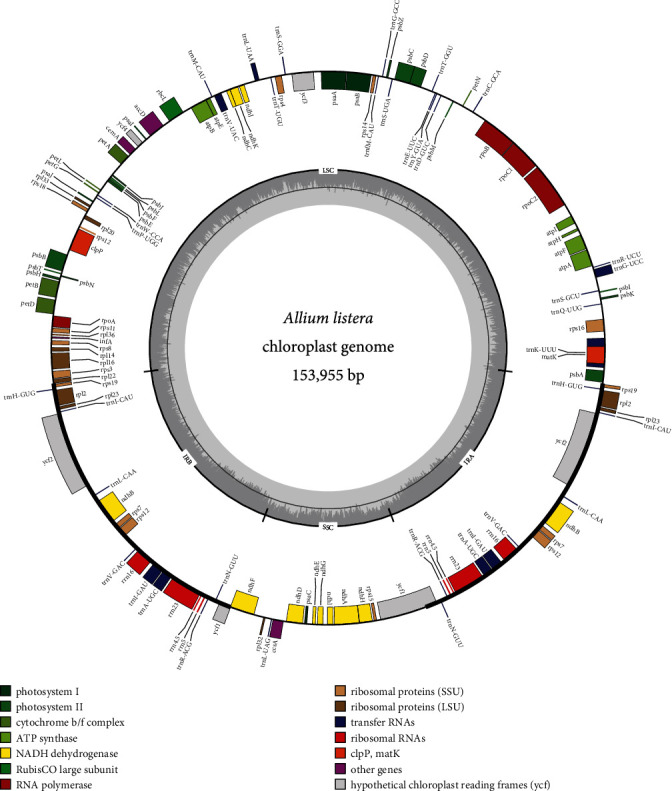
Plastid genome map of *A. listera*.

The plastomes of the three subfamilies (Allioideae, Agapanthoideae, and Amaryllidoideae) were all single circular molecules with a typical quadripartite structure (Figure 1). The plastome size of the 21 Allioideae species was found to be 152748-155373 bp, which in Agapanthoideae was 157055 bp and in 14 Amaryllidoideae species was 157241 bp to 160099 bp. Plastome lengths of LSC in Allioideae were from 82166 bp (*A. fasciculatum*) to 83358 bp (*A. cyathophorum*) and in SSC were varied from 17660 bp (*A. listera*) to 18770 bp (*A. funckiifolium*), which in Agapanthoideae were 85203 bp (LSC) and 18114 bp (SSC) and in Amaryllidoideae were 85656-86584 bp (LSC) and 16435-18542 bp (SSC). The GC contents of plastomes in Allioideae, Agapanthoideae, and Amaryllidoideae were 36.8-37.1%, 37.5%, and 37.7-38.0%, respectively. The gene number of the three subfamilies was ranged from 131 to 137. The detailed statistical information of the plastome sequence is summarized in Table 1.

### 3.2. Contraction and Expansion of IRs and Sequence Divergence

We found that the chloroplast genomes of Amaryllidaceae plants were relatively conserved on the IR boundary but that there was diversity in the location of the four regions of the chloroplast genome of different subfamilies and different species. From Figure S1, we found that in the chloroplast genomes of all species in the three subfamilies, the junction line between the LSC region and the IRa region (LR line) generally traversed the *rpl22* gene or the intergenic region between the *rpl22* gene and the *rps19* gene. The junction line between the IRa and the SSC (RS line) was located in the region of the *ycf_like* gene in the genomes of all subfamily species (except *Narcissus poeticus*), but the position on the pseudogene was different. In addition, we also found that there were a certain number of species in the three subfamilies that existed overlapping regions between the *ycf1_like* gene and the *ndhF* gene, and the length of the overlap region was as high as 85 bp in *Allium fetisowii*. The junction line between the SSC and IRb (SR line) traversed the coding region of the *ycf1* gene, but the coordinate positions were different. The junction line between the IRb and LSC (RL line) of three subfamilies was located in the intergenic region between the *rps19* gene and the *psbA* gene but had different coordinate positions (Supplementary Figure S1). We used mVISTA to visualize the chloroplast genome sequence diversity of the 36 species. The results showed that species between different subfamilies had obvious differences both in the coding region and noncoding region of the chloroplast genome (Supplementary Figure S2). When comparing the chloroplast genomes of different species in the same subfamily, we found that there was a high degree of similarity between the whole sequences.

### 3.3. Repeat Element Analysis and Codon Usage

**Figure 2:**
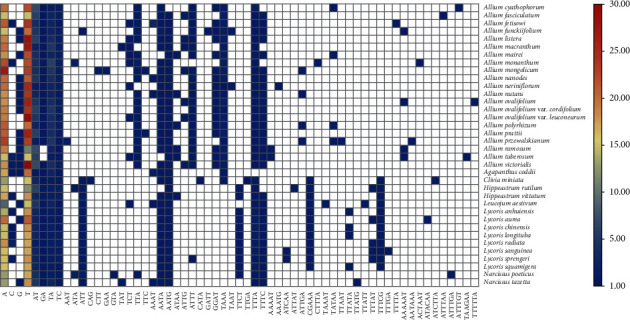
The number distribution of all types of SSR detected in 36 Amaryllidaceae plastid genomes. The result is shown with heat map using yellow as the intermediate transition color, from blue to red, while blue represents a low value, and red represents a high value.

**Figure 3:**
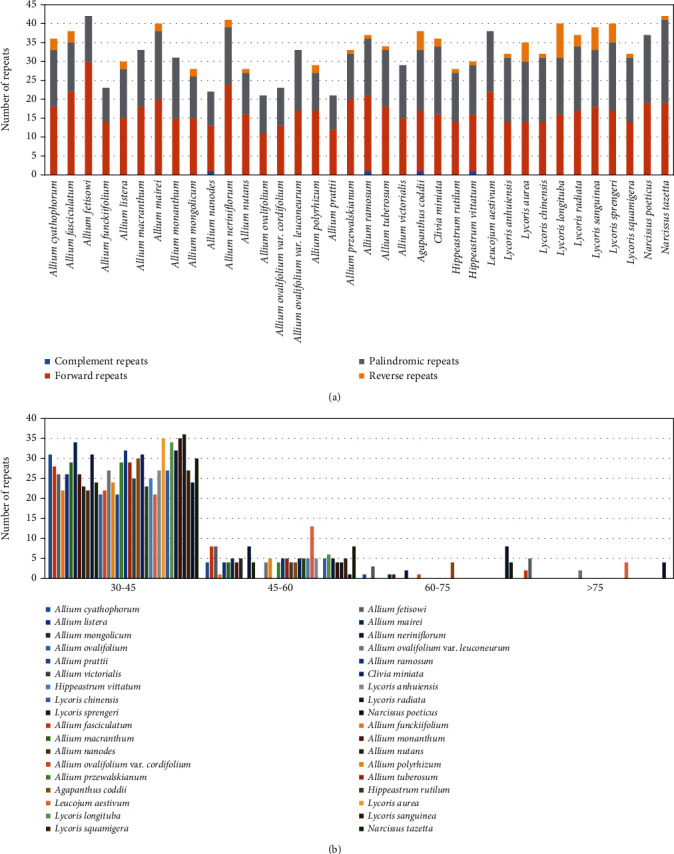
Analysis of repeat sequences in the 36 Amaryllidaceae plastid genomes. (a) Numbers of four repeat types. (b) Number of four types of repeats divided by length.

SSRs were detected in the three subfamilies (Supplementary Table S4). There were 1377 simple sequence repeats (SSRs) detected in 21 Allioideae species, and the most abundant type was mononucleotide repeats (65.6%), with other repeat types as follows: dinucleotides (17.1%), tetranucleotides (12.8%), trinucleotides (2.8%), pentanucleotides (1.0%) and hexanucleotides (0.7%). The above result was similar to the ratio of each component in the 717 SSRs detected in Amaryllidoideae, which only had three types of repeats in Agapanthoideae. For the 2144 SSRs detected in these 36 species, we performed relevant statistics on the types and numbers of their base combinations (Figure 2). Forward, palindromic, reverse, and complementary repeats in 36 plastomes were also detected (Supplementary Table S5). Among 21 Allioideae species, we detected 661 repeats 30-90 bp long, and the number of forward repeats (362) was higher than that of palindromic repeats (268), reverse repeats (20), and complement repeats (2). The four types of repeat ratios detected in Amaryllidoideae and Agapanthoideae were similar to the appeal results (Figure 3(a)). We divided all the repeats into four intervals according to length: 30-45 bp, 45-60 bp, 60-75 bp, and >75 bp. Among them, most of the repeats in Allioideae were 30-45 bp long (84.6%), followed by 45-60 bp (12.6%), 60-75 bp (1.4%), and >75 bp (1.4%) (Supplementary Table S6). The detected results in Amaryllidoideae and Agapanthoideae were consistent with those in Allioideae (Figure 3(b)).

**Figure 4:**
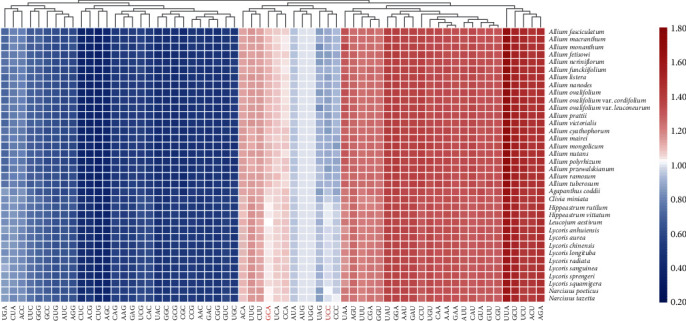
The RSCU values of all merged protein-coding genes for 36 Amaryllidaceae plastid genomes. The result is shown with heat map using the red values to indicate higher RSCU values and the blue values to indicate lower RSCU values.

**Figure 5:**
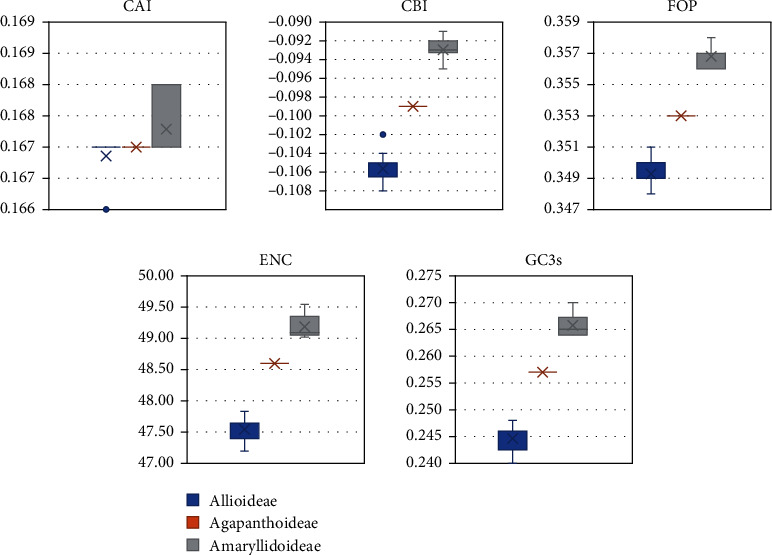
The comparative analysis of codon usage bias in three subfamilies species of Amaryllidaceae. CAI: codon adaptation index; CBI: codon bias index; FOP: frequency of optimal codons index; ENC: effective number of codons; GC3s: GC of synonymous codons in 3rd position.

We detected the CDS of the 36 plastomes separately, and six values were used to estimate the degree of preference for codons. The results of the RSCU values for all codons are shown in heat maps (Figure 4), which showed that most of the codon usage preferences remained at a consistent level in the three subfamilies, approximately half of the codons were used more frequently (RSCU > 1), and only two codons (ATG and TGG) had no bias (RSCU = 1). After statistical analysis, the other five parameters were displayed with box plots (Figure 5). We found that these five parameters had significant differences in the three subfamilies and Allioideae had the lowest correlation value among the five parameters, followed by Agapanthoideae and Amaryllidoideae (Supplementary Table S7).

### 3.4. Phylogenetic Relationships

**Figure 6:**
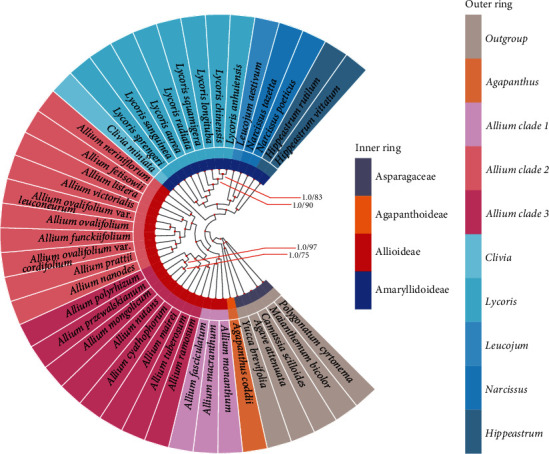
The phylogenetic relationships of 36 Amaryllidaceae species based on the whole plastid genomes. The phylogenetic tree is inferred from Bayesian inference (BI) and maximum likelihood (ML) analyses. Inconsistencies between PP and BS are marked separately at each node. Unmarked represents maximum support in both analyses.

**Figure 7:**
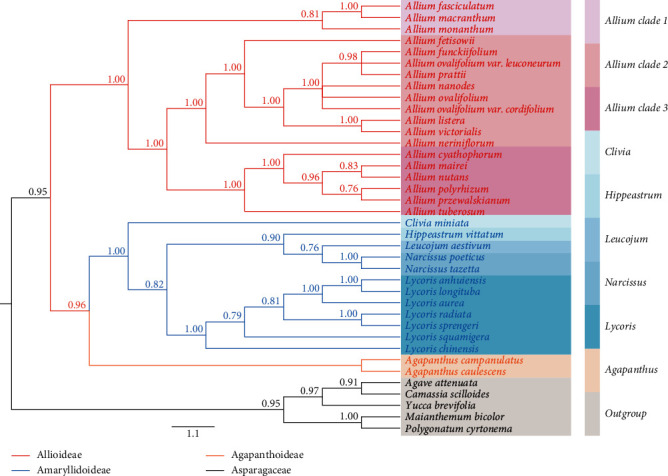
The phylogenetic relationships of 36 Amaryllidaceae species based on ITS. The phylogenetic tree is inferred from Bayesian inference (BI) and the posterior probabilities (PP) are marked separately at each node. Subfamilies of each species belong to, color of the bar is consistent with the species color.

We referred to the tree built with the chloroplast data as the CP tree. The CP trees reconstructed using the above two methods (ML and BI) were topologically consistent with each other (Figure 6), and there was little difference in well-supported branches in terms of bootstrap support values of ML (BS) or posterior probabilities of BI (PP). There was strong support for the monophyly of each family which was revealed based on shared SCG data (Figure 6). Amaryllidoideae was supported to be the sister of Allioideae, and Agapanthus coddii from Agapanthoideae had strong support to be sister to Allioideae and Amaryllidoideae (Figure 6). The ITS tree (Figure 7 and Supplementary Figure 3) was roughly comparable to the CP tree regarding subfamilies and intergeneric relationships but was weakly supported regarding interspecies and had some inconsistencies.

### 3.5. Selective Pressure Analysis

Based on the above results, we conducted a further positive selection analysis on the three subfamilies. Sixty-five protein-coding genes were initially considered for the positive selection analysis, and 60 of them were eventually selected after filtering (Supplementary Table S8). All genes detected with positive selection sites are listed in Table 2. For Allioideae species, all *p* values were insignificant in each gene range. However, 11 protein-coding genes (*atp8*, *atpF*, *accD*, *rps3*, *rps18*, *rpl16*, *petA*, *petG*, *psbE*, *psbJ*, and *ndhK*) were found with significant posterior probabilities in the BEB test, which means existing sites had positive selection (Table 3). In Amaryllidoideae and Agapanthoideae, there were 15 (*atpB*, *atpE*, *ndhD*, *ndhH*, *ndhI*, *ndhJ*, *petB*, *psbF*, *rpl22*, *rpl33*, *rps3*, *rps8*, *rps14*, *rps16*, and *ccsA*) and 12 (*ndhF*, *ndhH*, *petL*, *psbD*, *rpl20*, *rpl22*, *rpoA*, *rpoC2*, *rps3*, *rps4*, *rps8*, and *clpP*) similar genes, respectively (Supplementary Tables S9, S10). Among these protein-coding genes, most had only one positive selective site (*ndhK*, *petG*, *atpF*, etc.); some of them have more than one positive selective site, such as *petA* (seven sites) and *atpB* (nine sites) in Allioideae, *rpl20* (four sites) and *rpoA* (seven sites) in Agapanthoideae, and *rpl22* (two sites) and *ndhD* (three sites) in Amaryllidoideae.

### 3.6. Ancestral Character-State Reconstructions

**Figure 8:**
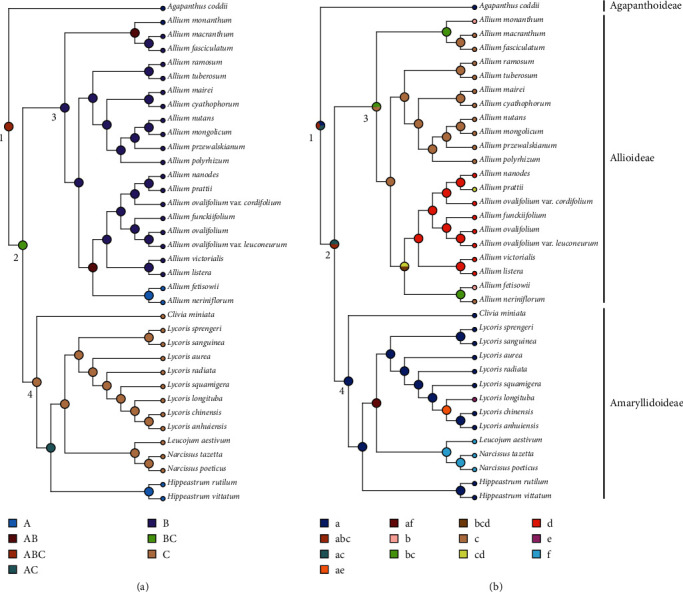
The ancestral character-state reconstructions of 36 Amaryllidaceae species. (a) The ancestral character-state reconstructions based on bulb types. (b) The ancestral character-state reconstructions based on leaf types.

Specific information and numbering for the two traits of Amaryllidaceae species is presented in Supplementary Table 11, and the traits reconstruction were presented in Figure 8. For bulbs, the results from RASP proposed one possible evolutionary route for Amaryllidaceae bulbs. The most recent common ancestor (MRCA) of Amaryllidaceae probably had spherical, ovoid, and cylindrical bulbs at the same time in different habitats, and the MRCA of Allioideae and Amaryllidoideae differentiated into cylindrical bulbs and ovoid bulbs. For leaves, in the possible evolutionary route for Amaryllidaceae leaves proposed by RASP, the MRCA of them may have appeared phenotype with many scales. This may also have been the case in the ancestors of the Allioideae and in the ancestors of the Allioideae and Amaryllidoideae. Within the Amaryllidoideae species, their MRCA may only have a ribbon leaf type and then differentiate into various leaf types, including ribbons, bars, and lanceolates. The information for pivotal nodes 1-4 that represent important ancestors of three subfamilies is marked in Figure 8 with numbers in black font.

## 4. Discussion

Currently, plastome data have been used to evaluate genetic variation in different orders, such as *Pilostyles*, *Salvia*, *Leguminosae*, and *Dipsacales* [45, 62–64]. The plastome sizes of all tested species varied from 152748 to 160099 bp, which was consistent with the length of most angiosperms [65]. It is striking that the plastome length of Amaryllidoideae and Agapanthoideae species was significantly longer than that of Allioideae species. Further statistics and comparison revealed that the difference in plastome length mainly results from the noncoding region length variation of LSC and SSC regions (Table 1), which is shorter in Allioideae species than in Amaryllidoideae and Agapanthoideae species. The results were in line with the widespread conservation that is characteristic of plastid genes (coding regions), especially photosynthesis-related genes [66], and has been reported in other plants [67]. Additionally, Amaryllidoideae species had the highest GC content not only in the whole chloroplast genome but also in the coding region and the noncoding region, followed by Agapanthoideae and Allioideae. Two reasons may explain this phenomenon: the selection of translation efficiency may result in a lack of G and C in the plastome [68, 69], and neutral mutation processes such as AT-biased gene conversion and AT-mutation pressure may cause lower GC content [70–72]. Similar results have been reported in other Allioideae species [11].

Large repeat sequences play an important role in sequence divergence and promote plastome rearrangement [73–75]. Here, we detected 1,199 long repeat sequences in the three subfamilies and found that the number of long repeat types was similar. Further analyses showed that most of the repeats are 30-45 bp, and the palindromic and forward types accounting for the largest proportion were similar to many other plastomes [76–78]. SSRs are considered to be potential resources in evolutionary studies and are effective in species discrimination and population genetic analyses exploring the biogeography of allied taxa [79–84]. From the SSR results, we found that some repeat types were specifically owned by Amaryllidoideae species, such as ATT, TTCT, CGAAA, and TTTCG, and some were possessed in Allioideae species, for example, TTA, ATTT, CGAT, and TAAA (Figure 2). These special SSRs can be used for the identification and classification of species within the Amaryllidaceae. Many SSRs have been detected and used for species identity and delimitation (e.g., *Lycoris*, *Psidium*, and *Asparagus*) [85–87]. Therefore, we believe that the repeat sequences detected in this study will provide useful information for studies of Amaryllidaceae in the future.

Codon usage is closely related to gene expression and natural selection pressure [88, 89]. From the results, we found that the phenomenon existed in all three subfamilies that 30 codons were used frequently (RSCU > 1) and all biased codons ended with a purine A or T. Codons that have a higher AT content are usually used in the plastomes, and the trend of using A/T in the third position of the codon is more obvious than using G/C [24, 90, 91]. Codons that encode leucine had the highest number, and the order of codon bias was TTA > CTT > TTG > CTA > CTC > CTG, which was consistent with the results found in other plants, such as *Ligusticum* and Geraniaceae [78, 92]. The codon GCA was found to be less used in Amaryllidoideae species than in the other two subfamilies, while TCC was more used in Amaryllidoideae species (Figure 4). From Figure 5, we found that five parameters involved in codon usage bias were lowest in Allioideae species, while Amaryllidoideae species had the highest values followed by Agapanthoideae (Figure 5). The calculated values revealed that the diverse codon usage patterns of different species may also be helpful for species identification and classification [93, 94].

Appropriate and multiple gene combinations are particularly important and efficient for accurate phylogenetic estimation. Nuclear ribosomal DNA genes (e.g., ITS and ETS), many cpDNA fragments (e.g., *rps16*, *matK*, and *trnL–trnF*), and chloroplast genomes have been used to infer the phylogeny of plants [12, 13, 17, 95, 96]. In this study, ML analysis and Bayesian inference were performed with two datasets (chloroplast SCGs and nrDNA ITS) to explore and reconstruct the phylogenetic relationships of Amaryllidaceae species. Our plastome analyses inferred well-supported relationships among the subfamily Amaryllidaceae (Figures 6 and 7). The monophyly and sisterhood of the three subfamilies was reconfirmed [12, 17, 97]. According to previous ITS-based studies, the *Allium* (Allioideae) species were divided into three evolutionary lineages (clade 1, clade 2, and clade 3) [17]. Here, our plastome phylogenomic analysis based on the SCGs provided strong support for the monophyly of *Allium* (Allioideae) and other Amaryllidaceae families (Figures 6 and 7, Supplementary Figure 3), which was in agreement with previous studies [12, 13, 17, 96, 98]. Besides, we further detected new species relationships within the three evolutionary lineages with high support values, including *Allium fasciculatum* in the first clade and *Allium funckiifolium*, *Allium listera*, *Allium ovalifolium* var. *cordifolium*, and *Allium ovalifolium* var. *leuconeurum* on the second clade. Previous studies performed the phylogenetic analysis of Amaryllidoideae using limited ITS or *matK* sequences and detected weaker support in phylogenetic relationships [99, 100]. Our plastome analysis based on SCGs revealed well-supported generic relationships inside Amaryllidoideae. Relationships among the five genera of Amaryllidoideae are well supported and generally in line with the previous studies [95, 97, 99–102]. Our ITS tree (Figure 7 and Supplementary Figure 3) provided strongly supported relationships among subfamilies of Amaryllidaceae and were highly consistent with the CP trees (Figure 6). However, the bootstrap support values of the ML tree among some genera and species were significantly lower than the posterior probability values of the BI tree. This may result from the use of different statistical inference methods. Relevant studies have shown that the BI method is more efficient, the node support rate in the BI method analysis results is higher than the corresponding results in other algorithms, and for closely related species sequences, the BI method works better [103–105]. All of the above results may indicate that the species relationships of Amaryllidaceae are complex. Although we detected some new species relationships and provided high support, relationships among species of Amaryllidaceae are still not well resolved (especially for species in *Lycoris* and in the third clade of Allioideae). In general, our plastome phylogenetic analysis reconstructed a well-supported tree for Amaryllidaceae and contributed to a better understanding of the Amaryllidaceae phylogeny. More extensive geographic information and genomic samples for further investigation are required in the future.

We conducted further selective pressure analysis on the three subfamilies. The 60 screened protein-coding genes of each subfamily were used to estimate the selective pressures, which may have evolved evolution to adapt to changing environmental conditions. Several genes were found to have significant posterior probabilities for codon sites under the BEB test in each of the three subfamilies, although the positive selection was insignificant in all genes (*p* value > 0.05), which may suggest they were under purifying selection (Table 3 and Supplementary Table S8 and S9). This result reflects the typical evolutionary conservation of plant plastid genes [106, 107]. Previous research has shown that codon sites with higher posterior probability can be regarded as positively selected sites, which means that genes possessing positively selected sites may be evolved under positive selection pressure [50]. Based on the above research results, it is worth noting that there are seven genes with positive selection sites related to photosynthesis in Allioideae, and eight and four similar genes were detected in Amaryllidoideae and Agapanthoideae.

Through further analysis, we found that these genes are associated with photosystem II subunits, subunits of NADH-dehydrogenase, subunits of the cytochrome b/f complex, and subunits of ATP synthase (Table 2). Photosystem II is the site of photosynthetic light reaction in plants, where integral membrane protein complexes use light energy to produce high-energy carriers ATP and NADPH [108–110]. Subunits of ATP synthase, subunits of NADH-dehydrogenase, and subunits of the cytochrome b/f complex are necessary for the generation of ATP in the electron transport chain [108, 111–113]. The genes mentioned above are all necessary for photosynthesis and participate in important physiological processes of plants [114]. These PSGs related to photosynthesis have been found in all three subfamilies, which may be closely related to the widespread distribution of Amaryllidaceae species on Earth [1]. Species of the three subfamilies are distributed in various environments, such as low temperature areas [58], temperate humid forest areas [15], hot arid and semiarid areas [115], and tropical grassland climate areas [116], and requirements for sufficient light for photosynthesis might have exerted strong selective forces on these genes, and in turn, these positively selected genes might contribute to species of the three subfamilies adapting various environment better. This phenomenon was also found in *Siraitia* and *Urophysa* genera [20, 117].

In addition, we also detected a series of genes related to self-replication in each subfamily. Plastid protein synthesis plays an essential role in plant development [118, 119]. Among the genes with positive selection sites, the *rpoA* gene has the most positive selection sites in Agapanthoideae, suggesting that the *rpoA* gene may play a pivotal role in the adaptive evolution of Agapanthoideae species. Studies have shown that plastid chromosomes encode four RNA polymerase genes, designated *rpoA*, *rpoB*, *rpoC1*, and *rpoC2* [120]. Notably, half of them (*rpoA* and *rpoC2*) were detected in selective pressure analysis within Agapanthoideae species. Both have been reported in Annonaceae and *Rehmannia* [121, 122]. The *rpoA* and *rpoC2* genes encode subunits *α* and *β*″ of plastid-encoded plastid RNA polymerase (PEP), respectively, which is believed to be a vital protein responsible for most photosynthetic gene expression [123]. In addition, the RNA polymerase *β*″ encoded by *rpoC2* may play an important role in the regulation of developmental pollination [117, 124]. The finding of these two genes under selective pressure indicated that they might be essential for growth and reproduction in Agapanthoideae. Gene *claP* encodes *clpP* proteases containing a gene family with six members (*claP1*-*claP6*) in *Arabidopsis* of the mustard family Brassicaceae [125]. It was only found under positive selection pressure in Agapanthoideae. The gene is detected in the chloroplast genome of all higher plants and is involved in various biological processes, ranging from plant growth changes to stress tolerance [125, 126]. It has been suggested that the *clpP* gene is essential for plant cell viability [127, 128], and the rapid evolution of the *claP* gene in Agapanthoideae species may help to adapt to its environment [129]. The accD gene related to the subunit of acetyl-CoA-carboxylase was only found in Allioideae with one positive selection site. Plastid *accD* is essential for plant leaf development or viability and fitness and has deep effects on leaf longevity and seed yield [130, 131]. It has been reported that *accD* gene shows an accelerated rate of evolution [65, 132, 133] and may be a useful marker for plastid evolution [134–136]. Allioideae species have many types of leaf morphology and physiological characteristics to adapt to different environments [96], and the *accD* gene may play an indispensable role in its adaptation process. We found the *ccsA* gene with one positive selection site in Amaryllidoideae, which encodes a protein that is required for heme attachment to C-type cytochrome and may be closely related to photosynthesis [137, 138]. It is generally present in land plants, while it is absent from the plastome of Physcomitrella patens [139].

In previous studies, most of the genes mentioned above have been reported under the pressure of positive selection [11, 140–142]. Species in Amaryllidaceae are mostly characterized by tunicate bulbs, rhizomes, or tubers and narrow linear basal leaves, but in different environments, many Amaryllidaceae species have evolved very different leaf and rhizome morphologies [98, 143]. The bulb and leaf are important taxonomic identifiers of Amaryllidaceae species, and they are also vital evidence and tools for species adaptation to various habitats [59, 60]. We reconstructed the evolution of bulb traits in Amaryllidaceae. The results show that their MRCA may have several types of bulblets, and then, the bulb type diverged in three subfamilies (Figure 8). *Allium* L. (Allioideae) is one of the largest genera of monocotyledons and is distributed in a variety of habitats including cliffs, shrubs, forests, and high-altitude grassy slopes [1, 15] They usually embed their entire bulbs between stone crevices and bush roots to hold themselves and absorb water [96]. *Allium* (Allioideae) species are dominated by slender cylindrical bulbs and usually have well-developed root systems, which may help them anchor themselves more easily (Figure 8). Through reconstructing the leaf traits, we found that the leaves of Agapanthoideae and Amaryllidoideae are generally differentiated into ribbons, while the leaves of Allioideae are mainly differentiated into two types, bar-shaped and oval. We found that all leaves that differentiated into oval leaves belonged to sect. *Anguinum* (marked by red shading), which were almost exclusively found in moist understory habitats [15, 96]. We speculate that the wide leaves may help *Anguinum* species utilize the weak light in the forest and transpiration more efficiently and then perform better photosynthesis [144–146]. These characteristics may be the key traits that will help them adapt to various harsh environments, such as severe cold, drought, saline soil, and high altitude, and enable them to produce and maintain a high level of plant diversity [147–149]. We suggest that these ecological characteristics of Amaryllidaceae reflect their remarkable adaptability to various environments due to diverse positive selection pressure on genes in the plastid, while most PSGs detected may play critical roles in the adaptation of plants in the Amaryllidaceae during the evolution process. Therefore, it is necessary to further investigate the important role of positive selection in the plastid genes of Amaryllidaceae species.

## 5. Conclusions

In this study, we investigated 36 complete chloroplast genomes of three Amaryllidaceae subfamily species. All chloroplast genomes exhibited a typical quadripartite structure and had highly similar genomic structures. SSRs, long repeats, and genes with positive selective sites were identified across the chloroplast genomes, which may be helpful for species identification or classification and can also be used as potential markers for phylogenetic investigations and population genetics studies. The monophyly of the three subfamilies was confirmed, and phylogenetic analysis showed that they are sisters to each other. Positive selection analysis identified some PSGs in each subfamily. These results provide a better understanding of the chloroplast genome characteristics in the three subfamilies, contributed to a better understanding of the Amaryllidaceae phylogeny, and afford more genomic information for further evolutionary investigations of Amaryllidaceae species.

## Figures and Tables

**Table 1:** Summary of the basic parameters from 36 Amaryllidaceae species plastid genomes.

Species	Total length		LSC length	SSC length	IR length	Gene number	Protein cording	rRNAs	tRNAs	Coding region		Non-coding region	
	Length (bp)	GC%								Length (bp)	GC%	Length (bp)	GC%
*Agapanthus coddii*	157055	37.5	85203	18114	26869	133	87	8	38	79029	37.9	78026	37.1
*Allium cyathophorum*	154174	36.8	83358	17882	26467	131	86	8	37	79383	37.3	74791	36.3
*Allium fasciculatum*	152931	37.1	82166	17837	26464	132	85	8	38	78936	37.5	73995	36.7
*Allium fetisowii*	154018	36.9	83202	17942	26437	132	86	8	38	79302	37.3	74716	36.5
*Allium funckiifolium*	155373	37.1	82813	18770	26895	132	87	8	37	79557	37.6	75816	36.6
*Allium listera*	153955	37.0	83259	17660	26518	132	87	8	37	79125	37.5	74830	36.5
*Allium macranthum*	152748	37.1	82541	17993	26107	132	86	8	38	78621	37.6	74127	36.6
*Allium mairei*	152913	36.9	82232	18141	26270	132	86	8	38	78915	37.3	73998	36.5
*Allium monanthum*	154730	37.0	83834	18008	26444	132	86	8	38	79308	37.5	75422	36.5
*Allium mongolicum*	153667	36.8	82644	18043	26490	132	87	8	38	79593	37.2	74074	36.4
*Allium nanodes*	153526	37.0	82519	17975	26516	132	87	8	37	79113	37.5	74413	36.5
*Allium neriniflorum*	154280	37.0	83130	18192	26479	132	86	8	38	79536	37.4	74744	36.6
*Allium nutans*	153456	36.9	82532	17952	26486	132	86	8	38	79237	37.3	74219	36.5
*Allium ovalifolium*	153713	37.0	82806	17933	26487	132	87	8	37	79179	37.5	74534	36.5
*Allium ovalifolium* var. *cordifolium*	153511	37.0	82451	18020	26520	132	87	8	37	79116	37.5	74395	36.5
*Allium ovalifolium* var. *leuconeurum*	153024	37.0	82261	17817	26473	132	87	8	37	78690	37.5	74334	36.5
*Allium polyrhizum*	152984	36.9	82437	17955	26296	132	86	8	38	79026	37.3	73958	36.5
*Allium prattii*	153516	37.0	82571	17971	26487	132	87	8	37	79158	37.4	74358	36.6
*Allium przewalskianum*	153509	36.9	82301	17718	26745	135	88	8	39	79926	37.2	73583	36.6
*Allium ramosum*	154034	36.9	83089	17907	26519	135	87	8	37	78966	37.4	75068	36.4
*Allium tuberosum*	154056	36.9	83067	17959	26515	131	88	8	39	78846	37.4	75210	36.4
*Allium victorialis*	154272	37.0	83322	17880	26535	132	87	8	37	79110	37.5	75162	36.5
*Clivia miniata*	158114	38.0	86203	18335	26788	133	87	8	38	79455	38.4	78659	37.6
*Hippeastrum rutilum*	158357	37.9	86450	18273	26817	133	87	8	38	79470	38.2	78887	37.6
*Hippeastrum vittatum*	158082	37.9	86165	18285	26816	133	87	8	38	79401	38.4	78681	37.4
*Leucojum aestivum*	157241	37.9	85656	18181	26702	133	86	8	38	79236	38.3	78005	37.5
*Lycoris anhuiensis*	158490	37.8	86464	18498	26764	135	87	8	38	79578	38.3	78912	37.3
*Lycoris aurea*	158690	37.7	86584	18542	26782	132	86	8	38	79239	38.3	79451	37.1
*Lycoris chinensis*	158484	37.8	86458	18498	26764	135	87	8	38	79578	38.3	78906	37.3
*Lycoris longituba*	158633	37.8	86461	18372	26900	136	85	8	38	78441	38.3	80192	37.3
*Lycoris radiata*	158436	37.7	86582	18234	26810	137	85	8	38	78873	38.2	79563	37.2
*Lycoris sanguinea*	158761	37.7	86528	18431	26901	137	86	8	38	79146	38.3	79615	37.1
*Lycoris sprengeri*	158747	37.7	86484	18479	26892	137	86	8	38	79137	38.3	79610	37.1
*Lycoris squamigera*	158459	37.8	86430	18501	26764	133	87	8	38	79554	38.2	78905	37.4
*Narcissus poeticus*	160099	37.8	86444	16435	28610	137	86	8	38	81995	38.4	78104	37.2
*Narcissus tazetta*	159376	38.0	85940	16452	28492	133	86	8	38	81261	38.4	78115	37.6

**Table 2:** List of 38 plastid coding genes with positive selection sites detected in three subfamilies.

Category	Group	Allioideae	Amaryllidoideae	Agapanthoideae
Self-replication	Large subunit of ribosome (LSU)	*rpl16*	*rpl22*	*rpl20*
			*rpl33*	*rpl22*
	Small subunit of ribosome (SSU)	*rps3* *rps18*	*rps3*	*rps3* *rps4* *rps8*
			*rps8*	
			*rps14*	
			*rps16*	
	DNA-dependent RNA polymerase			*rpoA*
				*rpoC2*
Photosynthesis	Photosystem II	*psbE*	*psbF*	*psbD*
		*psbJ*		
	Subunits of NADH-dehydrogenase	*ndhK*	*ndhD*	*ndhF* *ndhH*
			*ndhH*	
			*ndhI*	
			*ndhJ*	
	Subunits of cytochrome b/f complex	*petA*	*petB*	*prtL*
		*petG*		
	Subunits of ATP synthase	*atp8*	*atpB*	
		*atpF*	*atpE*	
Other genes	Subunit of acetyl-CoA-carboxylase	*accD*		
	C-type cytochrome synthesis gene		*ccsA*	
	ATP-dependent protease subunit p gene			*claP*

**Table 3:** The potential positive selection test based on the branch-site model in Allioideae.

Gene name	Null hypothesis			Alternative hypothesis			Significance test	
	lnL	df	Omega (w = 1)	lnL	df	Omega (w > 1)	BEB	*p* value
*petA*	-1979.00	74	1	-1978.91	75	9.50	30, T, 0.525; 43, G, 0.518; 92, L, 0.567; 138, Q, 0.567; 177, H, 0.569; 216, R, 0.527; and 238, V, 0.543	0.68
*petN*	-142.28	74	1	-142.28	75	1.00		1.00
*atpI*	-415.76	74	1	-415.76	75	1.00		1.00
*rpl33*	-454.52	74	1	-454.52	75	1.00		1.00
*rps11*	-957.14	74	1	-957.14	75	1.00		1.00
***rps3***	-1693.51	74	1	-1693.51	75	1.00	112, L, 0.552 and 125, H, 0.545	1.00
*psbH*	-418.26	74	1	-418.26	75	1.00		0.99
*rpl20*	-922.89	74	1	-922.89	75	1.00		1.00
*rpl14*	-822.07	74	1	-822.07	75	1.00		1.00
*ycf3*	-1124.46	74	1	-1124.46	75	1.35		0.97
*psbI*	-221.02	74	1	-221.02	75	1.00		1.00
*atpH*	-457.92	74	1	-457.92	75	1.00		1.00
*psaA*	-4212.68	74	1	-4212.68	75	1.00		1.00
*rpoA*	-2506.15	74	1	-2506.15	75	1.00		1.00
*ndhA*	-3318.09	74	1	-3318.09	75	1.00		1.00
*clpP*	-1136.93	74	1	-1136.93	75	1.00		1.00
*psbT*	-190.01	74	1	-190.01	75	1.00		1.00
***ndhK***	-1585.54	74	1	-1585.30	75	8.93	209, T, 0.778;	0.49
*ndhI*	-1379.34	74	1	-1379.34	75	1.00		1.00
***rps18***	-598.66	74	1	-598.66	75	1.00	27, R, 0.628 and 94, T, 0.620	1.00
*ndhG*	-1467.01	74	1	-1467.01	75	1.00		1.00
*psbA*	-2040.58	74	1	-2040.58	75	1.00		1.00
*psbN*	-229.94	74	1	-229.94	75	1.00		1.00
***petG***	-191.33	74	1	-191.21	75	1.00	5, F, 0.511	0.62
*ndhH*	-3117.69	74	1	-3117.69	75	1.00		1.00
*petL*	-164.26	74	1	-164.26	75	1.00		1.00
*rps4*	-1222.64	74	1	-1222.64	75	1.00		1.00
*ycf4*	-1157.74	74	1	-1157.74	75	1.00		1.00
*rps16*	-527.45	74	1	-527.45	75	1.00		1.00
*rbcL*	-3127.75	74	1	-3127.75	75	1.00		1.00
*atpA*	-3270.90	74	1	-3270.90	75	1.00		1.00
***atpB***	-3017.52	74	1	-3017.48	75	1	6, T, 0.577 and 7, T, 0.591	0.78
*ndhJ*	-977.72	74	1	-977.72	75	1		1
*rpoC2*	-10713.05	74	1	-10713.05	75	1		0.99
***atpF***	-1010.66	74	1	-1010.66	75	17.1	62, Y, 0.821	0.23
*psaJ*	-256.94	74	1	-256.94	75	1		1
*rpl36*	-238.98	74	1	-238.98	75	2.95		1.00
*rpoC1*	-4660.89	74	1	-4660.89	75	1.00		0.98
*ndhD*	-4280.68	74	1	-4280.68	75	1.00		1.00
*psbB*	-3140.03	74	1	-3140.03	75	1.00		1.00
*petD*	-969.28	74	1	-969.28	75	1.00		1.00
*psbF*	-195.99	74	1	-195.99	75	2.12		1.00
*rps14*	-602.61	74	1	-602.61	75	1.00		1.00
*rps8*	-889.08	74	1	-889.08	75	1.00		1.00
*psbC*	-2691.25	74	1	-2691.25	75	1.00		1.00
*ndhE*	-747.66	74	1	-747.66	75	1.07		0.99
*ndhF*	-7474.14	74	1	-7474.14	75	1.00		1.00
*rpl22*	-1203.35	74	1	-1203.35	75	1.00		1.00
*psaC*	-527.13	74	1	-527.13	75	1.00		1.00
*rpoB*	-6907.35	74	1	-6907.35	75	1.00		1.00
*ndhC*	-655.94	74	1	-655.94	75	1.00		1.00
*psaB*	-4158.16	74	1	-4158.16	75	1.00		1.00
***psbE***	-456.27	74	1	-456.27	75	1.00	11, A, 0.558	1.00
***rpl16***	-1062.20	74	1	-1062.20	75	1.00	127, R, 0.620	1.00
***accD***	-3439.19	74	1	-3439.19	75	1.00	26, N, 0.661	1.00
***psbJ***	-228.51	74	1	-228.47	75	1.51	25, I, 0.674 and 27, I, 0.660	0.79
*ccsA*	-3188.17	74	1	-3188.17	75	1.00		1.00
*psbD*	-2008.17	74	1	-2008.17	75	1.00		1.00
*atpE*	-900.46	74	1	-900.46	75	1.00		1.00
*petB*	-1237.44	74	1	-1237.44	75	1.00		1.00

Bold types are genes with positively selected sites. BEB: Bayesian empirical Bayes.

## Data Availability

The assembled plastid genome sequences of the 18 *Allium* species used in this study are available at the National Center for Biotechnology Information (http://nih.gov/). The accession number are as follows: MK820611 (*A. cyathophorum*), MK251467 (*A. fasciculatum*), MK820612 (*A. fetisowii*), MZ826268 (*A. funckiifolium*), MZ826269 (*A. listera*), MK820614 (*A. macranthum*), MK820615 (*A. mairei*), MH748538 (*A. monanthum*), MK820616 (*A. nanodes*), MK820617 (*A. neriniflorum*), MH341457 (*A. ovalifolium*), MZ826270 (*A. ovalifolium* var. *cordifolium*), MH341455 (*A. ovalifolium var. leuconeurum*), MK820618 (*A. polyrhizum*), MG739457 (*A. prattii*), MK820619 (*A. przewalskianum*), MK820623 (*A. tuberosum*), and MH341458 (*A. victorialis*).

## Supplementary Materials

Supplementary Materials Figure S1: comparison of the border regions among the 36 Amaryllidaceae plastid genomes. Figure S2: VISTA-based sequence identity plot of the 36 Amaryllidaceae plastid genomes using *Allium fasciculatum* as a reference. Figure S3: ML tree based on ITS. Table S1: information and GenBank accessions for sample collection. Table S2: the GenBank accessions of all 41 taxa plastome sequences used this study. Table S3: the GenBank accessions of all 38 taxa ITS sequences used this study. Table S4: number of six SSR types detected in 36 plastid genomes of 36 Amaryllidaceae species. Table S5: number of four repeat types in the plastid genomes of 36 Amaryllidaceae species. Table S6: frequency of four repeat types according to length in 36 Amaryllidaceae species. Table S7: codon usage table contains 14 parameters from 36 plastid genomes of Amaryllidaceae species. Table S8: the 65 protein-coding genes. Table S9: the potential positive selection test based on the branch-site model in Amaryllidoideae. Table S10: the potential positive selection test based on the branch-site model in Agapanthoideae. Table S11: information for two traits of 36 Amaryllidaceae species.
