# Supplementary material for: Comparative Plastome Analysis of Three Amaryllidaceae Subfamilies: Insights into Variation of Genome Characteristics, Phylogeny, and Adaptive Evolution
Source: Biomed Res Int. 2022 Mar 24;2022:3909596. doi: 10.1155/2022/3909596 (PMC8970886; doi:10.1155/2022/3909596)
Supplement: Supplementary Materials — Figure S1: comparison of the border regions among the 36 Amaryllidaceae plastid genomes. Figure S2: VISTA-based sequence identity plot of the 36 Amaryllidaceae plastid genomes using Allium fasciculatum as a reference. Figure S3: ML tree based on ITS. Table S1: information and GenBank accessions for sample collection. Table S2: the GenBank accessions of all 41 taxa plastome sequences used this study. Table S3: the GenBank accessions of all 38 taxa ITS sequences used this study. Table S4: number of six SSR types detected in 36 plastid genomes of 36 Amaryllidaceae species. Table S5: number of four repeat types in the plastid genomes of 36 Amaryllidaceae species. Table S6: frequency of four repeat types according to length in 36 Amaryllidaceae species. Table S7: codon usage table contains 14 parameters from 36 plastid genomes of Amaryllidaceae species. Table S8: the 65 protein-coding genes. Table S9: the potential positive selection test based on the branch-site model in Amaryllidoideae. Table S10: the potential positive selection test based on the branch-site model in Agapanthoideae. Table S11: information for two traits of 36 Amaryllidaceae species. [file 3909596.f1.zip › FigureS2 (1).pdf]

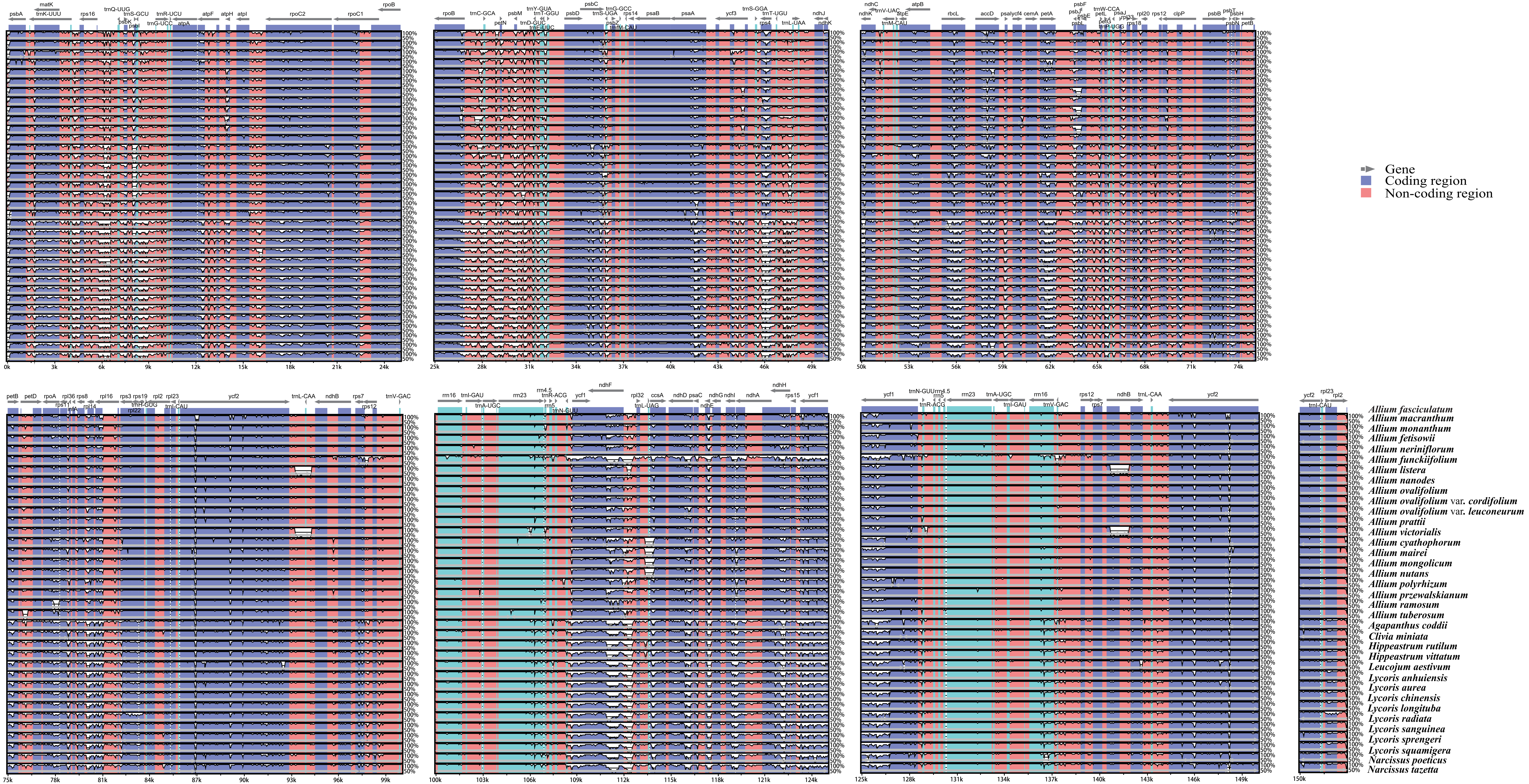
Gene
Coding region
Non-coding region
Allium fasciculatum
Allium macranthum
Allium monanthum
Allium fetisowii
Allium neriniflorum
Allium funckiifolium
Allium listera
Allium nanodes
Allium ovalifolium
Allium ovalifolium var. cordifolium
Allium ovalifolium var. leuconeurum
Allium prattii
Allium victorialis
Allium cyathophorum
Allium mairei
Allium mongolicum
Allium nutans
Allium polyrhizum
Allium przewalskianum
Allium ramosum
Allium tuberosum
Agapanthus coddii
Clivia miniata
Hippeastrum rutilum
Hippeastrum vittatum
Leucojum aestivum
Lycoris anhuiensis
Lycoris aurea
Lycoris chinensis
Lycoris longituba
Lycoris radiata
Lycoris sanguinea
Lycoris sprengeri
Lycoris squamigera
Narcissus poeticus
Narcissus tazetta
