# Supplementary material for: Comparative Plastome Analysis of Three Amaryllidaceae Subfamilies: Insights into Variation of Genome Characteristics, Phylogeny, and Adaptive Evolution
Source: Biomed Res Int. 2022 Mar 24;2022:3909596. doi: 10.1155/2022/3909596 (PMC8970886; doi:10.1155/2022/3909596)
Supplement: Supplementary Materials — Figure S1: comparison of the border regions among the 36 Amaryllidaceae plastid genomes. Figure S2: VISTA-based sequence identity plot of the 36 Amaryllidaceae plastid genomes using Allium fasciculatum as a reference. Figure S3: ML tree based on ITS. Table S1: information and GenBank accessions for sample collection. Table S2: the GenBank accessions of all 41 taxa plastome sequences used this study. Table S3: the GenBank accessions of all 38 taxa ITS sequences used this study. Table S4: number of six SSR types detected in 36 plastid genomes of 36 Amaryllidaceae species. Table S5: number of four repeat types in the plastid genomes of 36 Amaryllidaceae species. Table S6: frequency of four repeat types according to length in 36 Amaryllidaceae species. Table S7: codon usage table contains 14 parameters from 36 plastid genomes of Amaryllidaceae species. Table S8: the 65 protein-coding genes. Table S9: the potential positive selection test based on the branch-site model in Amaryllidoideae. Table S10: the potential positive selection test based on the branch-site model in Agapanthoideae. Table S11: information for two traits of 36 Amaryllidaceae species. [file 3909596.f1.zip › Table S1 (1).pdf]

Table S1 Information and Genbank accessions for sample collection

Species	Sequence Type	Sampling locality	Alt. (m)	NCBI accessions
Allium cyathophorum	complete chloroplast genome	Mangkang, Xizang province	3794	MK820611
Allium fasciculatum	complete chloroplast genome	Lasa, Xizang province	3650	MK251467
Allium fetisowi	complete chloroplast genome	Tacheng, Xinjiang province	1100	MK820612
Allium funckiifolium	complete chloroplast genome	Wushan, Chongqin Province	2400	MZ826268
Allium listera	complete chloroplast genome	Songxian, Henan Province	2216	MZ826269
Allium macranthum	complete chloroplast genome	Deqin, Yunnan province	3470	MK820614
Allium mairei	complete chloroplast genome	Xianggelila, Yunnan province	2852	MK820615
Allium monanthum	complete chloroplast genome	Jiamusi, Heilongjiang province	1274	MH748538
Allium nanodes	complete chloroplast genome	Daocheng, Sichuan province	4676	MK820616
Allium neriniflorum	complete chloroplast genome	Huairou, Beijing	1012	MK820617
Allium ovalifolium	complete chloroplast genome	Maerkang, Sichuan Province	2460	MH341457
Allium ovalifolium* var. *cordifolium	complete chloroplast genome	Maerkang, Sichuan Province	2510	MZ826270
Allium ovalifolium* var. *leuconeurum	complete chloroplast genome	Maerkang, Sichuan Province	2460	MH341455
Allium polyrhizum	complete chloroplast genome	Hami, Xinjiang province	1631	MK820618
Allium prattii	complete chloroplast genome	Litang, Sichuan province	1559	MG739457
Allium przewalskianum	complete chloroplast genome	Dawu, Qinghai province	3452	MK820619
Allium tuberosum	complete chloroplast genome	Emei Mountain, Sichuan province	1028	MK820623
Allium victorialis	complete chloroplast genome	Luanchuan, Henan province	2297	MH341458
