# Supplementary material for: Comparative Plastome Analysis of Three Amaryllidaceae Subfamilies: Insights into Variation of Genome Characteristics, Phylogeny, and Adaptive Evolution
Source: Biomed Res Int. 2022 Mar 24;2022:3909596. doi: 10.1155/2022/3909596 (PMC8970886; doi:10.1155/2022/3909596)
Supplement: Supplementary Materials — Figure S1: comparison of the border regions among the 36 Amaryllidaceae plastid genomes. Figure S2: VISTA-based sequence identity plot of the 36 Amaryllidaceae plastid genomes using Allium fasciculatum as a reference. Figure S3: ML tree based on ITS. Table S1: information and GenBank accessions for sample collection. Table S2: the GenBank accessions of all 41 taxa plastome sequences used this study. Table S3: the GenBank accessions of all 38 taxa ITS sequences used this study. Table S4: number of six SSR types detected in 36 plastid genomes of 36 Amaryllidaceae species. Table S5: number of four repeat types in the plastid genomes of 36 Amaryllidaceae species. Table S6: frequency of four repeat types according to length in 36 Amaryllidaceae species. Table S7: codon usage table contains 14 parameters from 36 plastid genomes of Amaryllidaceae species. Table S8: the 65 protein-coding genes. Table S9: the potential positive selection test based on the branch-site model in Amaryllidoideae. Table S10: the potential positive selection test based on the branch-site model in Agapanthoideae. Table S11: information for two traits of 36 Amaryllidaceae species. [file 3909596.f1.zip › Table S2 (1).pdf]

Table S2 The Genbank accessions of all 41 taxa plastome sequences used this study

**Species**	**NCBI accessions**
Agapanthus coddii	KX790363
Allium cyathophorum	MK820611
Allium fasciculatum	MK251467
Allium fetisowi	MK820612
Allium funckiifolium	MZ826268
Allium listera	MZ826269
Allium macranthum	MK820614
Allium mairei	MK820615
Allium monanthum	MH748538
Allium mongolicum	MT130396
Allium nanodes	MK820616
Allium neriniflorum	MK820617
Allium nutans	LT799837
Allium ovalifolium	MH341457
Allium ovalifolium* var. *cordifolium	MZ826270
Allium ovalifolium* var. *leuconeurum	MH341455
Allium polyrhizum	MK820618
Allium prattii	MG739457
Allium przewalskianum	MK820619
Allium ramosum	MH159131
Allium tuberosum	MK820623
Allium victorialis	MH341458
Clivia miniata	MN857162
Hippeastrum rutilum	MT133568
Hippeastrum vittatum	MT762362
Leucojum aestivum	MH422130
Lycoris anhuiensis	MT700550
Lycoris aurea	MN831471
Lycoris chinensis	MT700549
Lycoris longituba	MK353218
Lycoris radiata	MK353219
Lycoris sanguinea	MK353220
Lycoris sprengeri	MK353221
Lycoris squamigera	MH118290
Narcissus poeticus	MH706763
Narcissus tazetta	MN432153
Agave attenuata	MT083827
Camassia scilloides	KP008322
Maianthemum bicolor	KY908527
Polygonatum cyrtonema	MH808014
Yucca brevifolia	MF964125

The species sequenced by ourselves have been marked in bold
