# Supplementary material for: Comparative Plastome Analysis of Three Amaryllidaceae Subfamilies: Insights into Variation of Genome Characteristics, Phylogeny, and Adaptive Evolution
Source: Biomed Res Int. 2022 Mar 24;2022:3909596. doi: 10.1155/2022/3909596 (PMC8970886; doi:10.1155/2022/3909596)
Supplement: Supplementary Materials — Figure S1: comparison of the border regions among the 36 Amaryllidaceae plastid genomes. Figure S2: VISTA-based sequence identity plot of the 36 Amaryllidaceae plastid genomes using Allium fasciculatum as a reference. Figure S3: ML tree based on ITS. Table S1: information and GenBank accessions for sample collection. Table S2: the GenBank accessions of all 41 taxa plastome sequences used this study. Table S3: the GenBank accessions of all 38 taxa ITS sequences used this study. Table S4: number of six SSR types detected in 36 plastid genomes of 36 Amaryllidaceae species. Table S5: number of four repeat types in the plastid genomes of 36 Amaryllidaceae species. Table S6: frequency of four repeat types according to length in 36 Amaryllidaceae species. Table S7: codon usage table contains 14 parameters from 36 plastid genomes of Amaryllidaceae species. Table S8: the 65 protein-coding genes. Table S9: the potential positive selection test based on the branch-site model in Amaryllidoideae. Table S10: the potential positive selection test based on the branch-site model in Agapanthoideae. Table S11: information for two traits of 36 Amaryllidaceae species. [file 3909596.f1.zip › Table S3 (1).pdf]

Table S3 The Genbank accessions of all 38 taxa ITS sequences used this study

**Species**	**NCBI accessions**
Agapanthus campanulatus	JX464256
Agapanthus caulescens	AF373070
Allium cyathophorum	KP114566
Allium fasciculatum	GQ181068
Allium fetisowi	AF037619
Allium funckiifolium	HQ690557
Allium listera	GQ181063
Allium macranthum	GQ181072
Allium mairei	HQ690290
Allium monanthum	AJ412745
Allium nanodes	GU566615
Allium neriniflorum	HQ690560
Allium nutans	GQ181075
Allium ovalifolium	GQ181084
Allium ovalifolium var. *cordifolium*	GQ181085
Allium ovalifolium var. *leuconeurum*	GU566616
Allium polyrhizum	GQ181107
Allium prattii	HQ690537
Allium przewalskianum	HQ690283
Allium tuberosum	HE774703
Allium victorialis	GQ181082
Clivia miniata	AF324027
Hippeastrum vittatum	JX464307
Leucojum aestivum	AY101281
Lycoris anhuiensis	AY942711
Lycoris aurea	AY942710
Lycoris chinensis	AY563135
Lycoris longituba	AY942714
Lycoris radiata	AY942718
Lycoris sprengeri	AY942716
Lycoris squamigera	AY942728
Narcissus poeticus	MG197593
Narcissus tazetta	MG197586
Agave attenuata	MT083827
Camassia scilloides	KP008322
Maianthemum bicolor	KY908527
Polygonatum cyrtonema	MH808014
Yucca brevifolia	MF964125
