# Supplementary material for: Comparative Plastome Analysis of Three Amaryllidaceae Subfamilies: Insights into Variation of Genome Characteristics, Phylogeny, and Adaptive Evolution
Source: Biomed Res Int. 2022 Mar 24;2022:3909596. doi: 10.1155/2022/3909596 (PMC8970886; doi:10.1155/2022/3909596)
Supplement: Supplementary Materials — Figure S1: comparison of the border regions among the 36 Amaryllidaceae plastid genomes. Figure S2: VISTA-based sequence identity plot of the 36 Amaryllidaceae plastid genomes using Allium fasciculatum as a reference. Figure S3: ML tree based on ITS. Table S1: information and GenBank accessions for sample collection. Table S2: the GenBank accessions of all 41 taxa plastome sequences used this study. Table S3: the GenBank accessions of all 38 taxa ITS sequences used this study. Table S4: number of six SSR types detected in 36 plastid genomes of 36 Amaryllidaceae species. Table S5: number of four repeat types in the plastid genomes of 36 Amaryllidaceae species. Table S6: frequency of four repeat types according to length in 36 Amaryllidaceae species. Table S7: codon usage table contains 14 parameters from 36 plastid genomes of Amaryllidaceae species. Table S8: the 65 protein-coding genes. Table S9: the potential positive selection test based on the branch-site model in Amaryllidoideae. Table S10: the potential positive selection test based on the branch-site model in Agapanthoideae. Table S11: information for two traits of 36 Amaryllidaceae species. [file 3909596.f1.zip › Table S4 (1).pdf]

Table S4 Number of six SSR types detected in 36 plastid genomes of 36 Amaryllidaceae species

**Species**	**Hexa**	**Penta**	**Tetra**	**Tri**	**Di**	**Mono**
Agapanthus coddii	0	0	5	0	6	39
Allium cyathophorum	1	1	7	1	12	42
Allium fasciculatum	1	0	8	1	12	30
Allium fetisowi	0	1	11	2	9	46
Allium funckiifolium	1	0	11	2	11	36
Allium listera	0	0	9	2	13	48
Allium macranthum	0	0	7	2	11	47
Allium mairei	0	1	8	2	10	41
Allium monanthum	1	1	5	5	13	45
Allium mongolicum	0	1	9	4	12	50
Allium nanodes	0	0	9	1	12	42
Allium neriniflorum	0	1	8	1	8	46
Allium nutans	0	1	9	1	8	43
Allium ovalifolium	2	0	9	1	12	45
Allium ovalifolium* var. *cordifolium	0	0	7	1	14	44
Allium ovalifolium* var. *leuconeurum	0	0	9	2	12	48
Allium polyrhizum	0	2	9	1	10	41
Allium prattii	0	0	8	2	13	48
Allium przewalskianum	1	3	6	3	9	47
Allium ramosum	1	1	10	1	11	31
Allium tuberosum	2	1	8	2	9	34
Allium victorialis	0	0	9	1	14	50
Clivia miniata	1	2	6	2	7	29
Hippeastrum rutilum	0	4	3	2	11	27
Hippeastrum vittatum	0	2	6	1	8	36
Leucojum aestivum	0	4	5	2	10	25
Lycoris anhuiensis	0	3	6	1	7	33
Lycoris aurea	1	3	7	1	7	42
Lycoris chinensis	0	3	6	1	7	34
Lycoris longituba	0	3	6	1	8	33
Lycoris radiata	0	3	6	1	7	37
Lycoris sanguinea	0	5	6	1	8	33
Lycoris sprengeri	0	4	6	1	8	35
Lycoris squamigera	0	3	6	1	7	33
Narcissus poeticus	1	0	5	6	8	30
Narcissus tazetta	0	1	5	2	10	32
