# Supplementary material for: Comparative Plastome Analysis of Three Amaryllidaceae Subfamilies: Insights into Variation of Genome Characteristics, Phylogeny, and Adaptive Evolution
Source: Biomed Res Int. 2022 Mar 24;2022:3909596. doi: 10.1155/2022/3909596 (PMC8970886; doi:10.1155/2022/3909596)
Supplement: Supplementary Materials — Figure S1: comparison of the border regions among the 36 Amaryllidaceae plastid genomes. Figure S2: VISTA-based sequence identity plot of the 36 Amaryllidaceae plastid genomes using Allium fasciculatum as a reference. Figure S3: ML tree based on ITS. Table S1: information and GenBank accessions for sample collection. Table S2: the GenBank accessions of all 41 taxa plastome sequences used this study. Table S3: the GenBank accessions of all 38 taxa ITS sequences used this study. Table S4: number of six SSR types detected in 36 plastid genomes of 36 Amaryllidaceae species. Table S5: number of four repeat types in the plastid genomes of 36 Amaryllidaceae species. Table S6: frequency of four repeat types according to length in 36 Amaryllidaceae species. Table S7: codon usage table contains 14 parameters from 36 plastid genomes of Amaryllidaceae species. Table S8: the 65 protein-coding genes. Table S9: the potential positive selection test based on the branch-site model in Amaryllidoideae. Table S10: the potential positive selection test based on the branch-site model in Agapanthoideae. Table S11: information for two traits of 36 Amaryllidaceae species. [file 3909596.f1.zip › Table S5 (1).pdf]

Table S5 Number of four repeat types in the plastid genomes of 36 Amaryllidaceae species

**Species**	**Complement repeats**	**Forward repeats**	**Palindromic repeats**	**Reverse repeats**
Agapanthus coddii	1	16	16	5
Allium cyathophorum	0	18	15	3
Allium fasciculatum	0	22	13	3
Allium fetisowi	0	30	12	0
Allium funckiifolium	0	14	9	0
Allium listera	0	15	13	2
Allium macranthum	0	18	15	0
Allium mairei	0	20	18	2
Allium monanthum	0	15	16	0
Allium mongolicum	0	15	11	2
Allium nanodes	1	12	9	0
Allium neriniflorum	0	24	15	2
Allium nutans	0	16	11	1
Allium ovalifolium	0	11	10	0
Allium ovalifolium* var. *cordifolium	0	13	10	0
Allium ovalifolium* var. *leuconeurum	0	17	16	0
Allium polyrhizum	0	17	10	2
Allium prattii	0	12	9	0
Allium przewalskianum	0	20	12	1
Allium ramosum	1	20	15	1
Allium tuberosum	0	18	15	1
Allium victorialis	0	15	14	0
Clivia miniata	0	16	18	2
Hippeastrum rutilum	0	14	13	1
Hippeastrum vittatum	1	15	13	1
Leucojum aestivum	0	22	16	0
Lycoris anhuiensis	0	14	17	1
Lycoris aurea	0	14	16	5
Lycoris chinensis	0	14	17	1
Lycoris longituba	0	16	15	9
Lycoris radiata	0	17	17	3
Lycoris sanguinea	0	18	15	6
Lycoris sprengeri	0	17	18	5
Lycoris squamigera	0	14	17	1
Narcissus poeticus	0	19	18	0
Narcissus tazetta	0	19	22	1
