# Supplementary material for: Comparative Plastome Analysis of Three Amaryllidaceae Subfamilies: Insights into Variation of Genome Characteristics, Phylogeny, and Adaptive Evolution
Source: Biomed Res Int. 2022 Mar 24;2022:3909596. doi: 10.1155/2022/3909596 (PMC8970886; doi:10.1155/2022/3909596)
Supplement: Supplementary Materials — Figure S1: comparison of the border regions among the 36 Amaryllidaceae plastid genomes. Figure S2: VISTA-based sequence identity plot of the 36 Amaryllidaceae plastid genomes using Allium fasciculatum as a reference. Figure S3: ML tree based on ITS. Table S1: information and GenBank accessions for sample collection. Table S2: the GenBank accessions of all 41 taxa plastome sequences used this study. Table S3: the GenBank accessions of all 38 taxa ITS sequences used this study. Table S4: number of six SSR types detected in 36 plastid genomes of 36 Amaryllidaceae species. Table S5: number of four repeat types in the plastid genomes of 36 Amaryllidaceae species. Table S6: frequency of four repeat types according to length in 36 Amaryllidaceae species. Table S7: codon usage table contains 14 parameters from 36 plastid genomes of Amaryllidaceae species. Table S8: the 65 protein-coding genes. Table S9: the potential positive selection test based on the branch-site model in Amaryllidoideae. Table S10: the potential positive selection test based on the branch-site model in Agapanthoideae. Table S11: information for two traits of 36 Amaryllidaceae species. [file 3909596.f1.zip › Table S6 (1).pdf]

Table S6 Frequency of four repeat types according to length in 36 Amaryllidaceae species

Species	30-45	45-60	60-75	>75
Agapanthus coddii	30	4	4	0
Allium cyathophorum	31	4	1	0
Allium fasciculatum	28	8	0	2
Allium fetisowi	26	8	3	5
Allium funckiifolium	22	1	0	0
Allium listera	26	4	0	0
Allium macranthum	29	4	0	0
Allium mairei	34	5	1	0
Allium monanthum	26	4	1	0
Allium mongolicum	23	5	0	0
Allium nanodes	22	0	0	0
Allium neriniflorum	31	8	2	0
Allium nutans	24	4	0	0
Allium ovalifolium	21	0	0	0
Allium ovalifolium* var. *cordifolium	22	0	1	0
Allium ovalifolium* var. *leuconeurum	27	4	0	2
Allium polyrhizum	24	5	0	0
Allium prattii	21	0	0	0
Allium przewalskianum	29	4	0	0
Allium ramosum	32	5	0	0
Allium tuberosum	29	5	0	0
Allium victorialis	25	4	0	0
Clivia miniata	31	5	0	0
Hippeastrum rutilum	23	5	0	0
Hippeastrum vittatum	25	5	0	0
Leucojum aestivum	21	13	0	4
Lycoris anhuiensis	27	5	0	0
Lycoris aurea	35	0	0	0
Lycoris chinensis	27	5	0	0
Lycoris longituba	34	6	0	0
Lycoris radiata	32	5	0	0
Lycoris sanguinea	35	4	0	0
Lycoris sprengeri	36	4	0	0
Lycoris squamigera	27	5	0	0
Narcissus poeticus	24	1	8	4
Narcissus tazetta	30	8	4	0
