# Supplementary material for: Comparative Plastome Analysis of Three Amaryllidaceae Subfamilies: Insights into Variation of Genome Characteristics, Phylogeny, and Adaptive Evolution
Source: Biomed Res Int. 2022 Mar 24;2022:3909596. doi: 10.1155/2022/3909596 (PMC8970886; doi:10.1155/2022/3909596)
Supplement: Supplementary Materials — Figure S1: comparison of the border regions among the 36 Amaryllidaceae plastid genomes. Figure S2: VISTA-based sequence identity plot of the 36 Amaryllidaceae plastid genomes using Allium fasciculatum as a reference. Figure S3: ML tree based on ITS. Table S1: information and GenBank accessions for sample collection. Table S2: the GenBank accessions of all 41 taxa plastome sequences used this study. Table S3: the GenBank accessions of all 38 taxa ITS sequences used this study. Table S4: number of six SSR types detected in 36 plastid genomes of 36 Amaryllidaceae species. Table S5: number of four repeat types in the plastid genomes of 36 Amaryllidaceae species. Table S6: frequency of four repeat types according to length in 36 Amaryllidaceae species. Table S7: codon usage table contains 14 parameters from 36 plastid genomes of Amaryllidaceae species. Table S8: the 65 protein-coding genes. Table S9: the potential positive selection test based on the branch-site model in Amaryllidoideae. Table S10: the potential positive selection test based on the branch-site model in Agapanthoideae. Table S11: information for two traits of 36 Amaryllidaceae species. [file 3909596.f1.zip › TableS10.pdf]

Table S10 The potential positive selection test based on the branch-site model in Agapanthoideae

Gene name	Null hypothesis			Alternative hypothesis			Significance test	
	lnL	df	omega (w = 1)	lnL	df	omega (w > 1)	BEB	p-value
rpl36	-238.98299	74	1	-238.98299	75	1		1
psbT	-190.010337	74	1	-190.010337	75	1		1
rbcL	-3127.68561	74	1	-3127.68551	75	1		0.9889
atpE	-900.463345	74	1	-900.463344	75	1		0.9989
rpl33	-454.522941	74	1	-454.522941	75	1		1
rpl20	-921.544673	**74**	**1**	-920.12639	**75**	185.0805	48,Q,0.663;72,R,0.745;109,C,0.640;117,E,0.705;	0.09214
ndhI	-1379.24559	74	1	-1379.24559	75	1		1
psaA	-4212.47054	74	1	-4212.47054	75	1		1
atpH	-457.921491	**74**	**1**	-457.921491	**75**	2.07574		1
ndhK	-1585.55986	74	1	-1585.55986	75	1		1
petA	-1979.51587	74	1	-1979.51587	75	1		1
psbB	-3139.91094	74	1	-3139.88878	75	999		0.8332
ndhD	-4280.68323	74	1	-4280.68323	75	1.411		1
ndhC	-655.941572	74	1	-655.941572	75	2.38106		1
petL	-163.423269	74	1	-163.177194	75	100.26703	11,V,0.930;	0.483
psbF	-195.993517	74	1	-195.993517	75	2.13333		1
atpA	-3270.72074	74	1	-3270.72074	75	1		1
rpl22	-1203.26715	74	1	-1203.26715	75	1	27,M,0.556;74,S,0.518;	1
rps14	-602.606893	74	1	-602.606893	75	1		1
ycf4	-1157.74056	74	1	-1157.74056	75	1		1
psaJ	-256.9422	74	1	-256.9422	75	1		1
psbI	-221.015061	74	1	-221.015061	75	1		1
rps3	-1693.50986	74	1	-1693.50986	75	1	138,I,0.514;	1
petG	-191.33077	74	1	-191.33077	75	2.93812		1
psbD	-2005.96183	74	1	-2005.95446	75	1.38656	4,T,0.665;29,I,0.640;	0.9034
ndhF	-7469.83033	74	1	-7469.35719	75	6.47859	203,Y,0.639;250,V,0.551;368,I,0.561;431,F,0.940;475,T,0.606;522,V,0.551;541,A,0.563;543,Y,0.680;577,F,0.614;586,F,0.597;666,V,0.672;674,V,0.719;681,F,0.599;	0.3307
psbC	-2691.2631	74	1	-2691.2631	75	1		1
rpoA	-2504.75906	74	1	-2504.7535	75	1.2902	2,I,0.619;88,F,0.568;130,I,0.619;148,R,0.601;161,I,0.611;162,K,0.637;336,V,0.608;	0.916
rpoC1	-4660.88635	74	1	-4660.88635	75	1		1
rpl16	-1062.20017	74	1	-1062.20017	75	1		1
petN	-142.280065	74	1	-142.280065	75	1.59772		1
rps18	-599.161135	74	1	-599.161135	75	1		1
clpP	-1134.69333	74	1	-1134.71958	75	1	60,R,0.839;176,Y,0.842;188,N,0.851;	0.8188
psbJ	-229.518539	74	1	-229.518539	75	1		1
rpoB	-6907.34557	74	1	-6907.34557	75	1		0.997
rps11	-957.143577	74	1	-957.143577	75	1		1
ndhA	-3318.0857	74	1	-3318.0857	75	1		1
rps4	-1222.62061	74	1	-1222.62067	75	1	41,K,0.538;	0.991
psaC	-527.133471	74	1	-527.133485	75	2.3571		0.9958
rpl14	-822.066268	74	1	-822.066268	75	1		1
petD	-969.281459	74	1	-969.281459	75	1		1
accD	-3439.19393	74	1	-3439.19393	75	1		1
ndhH	-3117.68534	74	1	-3117.68534	75	1	282,A,0.521;	1
atpI	-415.758303	74	1	-415.758303	75	1		1
psbN	-229.938474	74	1	-229.938474	75	1		1
ndhE	-747.660014	74	1	-747.66009	75	1		0.9902
ndhJ	-977.72117	74	1	-977.72117	75	1		1
rpoC2	-10710.1123	74	1	-10709.9834	75	12.77308	440,F,0.512;507,K,0.516;514,T,0.510;545,K,0.558;559,D,0.542;583,N,0.540;685,I,0.513;817,R,0.502;864,Y,0.639;894,R,0.575;905,I,0.525;990,E,0.550;1014,S,0.516;1059,F,0.533;1077,N,0.520;1322,Y,0.518;1335,I,0.528	0.6115
atpB	-3019.66855	74	1	-3019.66855	75	1		1
ndhG	-1467.01117	74	1	-1467.01117	75	2.92866		1
psbA	-2040.57995	74	1	-2040.57995	75	1		1
atpF	-1010.66226	74	1	-1010.66226	75	1		1
rps8	-888.588266	74	1	-888.588266	75	1	21,K,0.508;55,F,0.565;56,F,0.577;89,F,0.565;110,Q,0.552;	1
petB	-1237.44418	74	1	-1237.44418	75	1		1
ccsA	-3178.85885	74	1	-3101.53418	75	1		1
psbH	-418.262901	74	1	-418.262901	75	1		1
psaB	-4158.15542	74	1	-4158.15542	75	2.95719		0.9984
ycf3	-1124.39618	74	1	-1124.39606	75	1		0.9875
rps16	-527.448993	74	1	-527.448994	75	2.8672		0.9989
psbE	-456.300584	74	1	-456.300584	75	1		1

Bold types are genes with positively selected sites. BEB, Bayesian Empirical Bayes.
