# Supplementary material for: Comparative Plastome Analysis of Three Amaryllidaceae Subfamilies: Insights into Variation of Genome Characteristics, Phylogeny, and Adaptive Evolution
Source: Biomed Res Int. 2022 Mar 24;2022:3909596. doi: 10.1155/2022/3909596 (PMC8970886; doi:10.1155/2022/3909596)
Supplement: Supplementary Materials — Figure S1: comparison of the border regions among the 36 Amaryllidaceae plastid genomes. Figure S2: VISTA-based sequence identity plot of the 36 Amaryllidaceae plastid genomes using Allium fasciculatum as a reference. Figure S3: ML tree based on ITS. Table S1: information and GenBank accessions for sample collection. Table S2: the GenBank accessions of all 41 taxa plastome sequences used this study. Table S3: the GenBank accessions of all 38 taxa ITS sequences used this study. Table S4: number of six SSR types detected in 36 plastid genomes of 36 Amaryllidaceae species. Table S5: number of four repeat types in the plastid genomes of 36 Amaryllidaceae species. Table S6: frequency of four repeat types according to length in 36 Amaryllidaceae species. Table S7: codon usage table contains 14 parameters from 36 plastid genomes of Amaryllidaceae species. Table S8: the 65 protein-coding genes. Table S9: the potential positive selection test based on the branch-site model in Amaryllidoideae. Table S10: the potential positive selection test based on the branch-site model in Agapanthoideae. Table S11: information for two traits of 36 Amaryllidaceae species. [file 3909596.f1.zip › TableS11.pdf]

Table S11 Information for two traits of 36 Amaryllidaceae species

**species**	**bulb types**	**bulb numbering**	**leaf types**	**leaf numbering**
Agapanthus coddii	spherical	A	ribbon	a
Allium monanthum	spherical	A	wide bar	b
Allium macranthum	cylindrical	B	bar	c
Allium fasciculatum	cylindrical	B	bar	c
Allium ramosum	cylindrical	B	bar	c
Allium tuberosum	cylindrical	B	bar	c
Allium mairei	cylindrical	B	bar	c
Allium cyathophorum	cylindrical	B	bar	c
Allium nutans	cylindrical	B	bar	c
Allium mongolicum	cylindrical	B	bar	c
Allium przewalskianum	cylindrical	B	bar	c
Allium polyrhizum	cylindrical	B	bar	c
Allium nanodes	cylindrical	B	oval	d
Allium prattii	cylindrical	B	bar, oval	cd
Allium ovalifolium* var. *cordifolium	cylindrical	B	oval	d
Allium funckiifolium	cylindrical	B	oval	d
Allium ovalifolium	cylindrical	B	oval	d
Allium ovalifolium* var. *leuconeurum	cylindrical	B	oval	d
Allium victorialis	cylindrical	B	oval	d
Allium listera	cylindrical	B	oval	d
Allium fetisowii	spherical	A	wide bar	b
Allium neriniflorum	spherical	A	bar	c
Clivia miniata	ovoid	C	ribbon	a
Lycoris sprengeri	ovoid	C	ribbon	a
Lycoris sanguinea	ovoid	C	ribbon	a
Lycoris aurea	ovoid	C	ribbon	a
Lycoris radiata	ovoid	C	ribbon	a
Lycoris squamigera	ovoid	C	ribbon	a
Lycoris longituba	ovoid	C	lanceolate	e
Lycoris chinensis	ovoid	C	ribbon	a
Lycoris anhuiensis	ovoid	C	ribbon	a
Leucojum aestivum	ovoid	C	wide line	f
Narcissus tazetta	ovoid	C	wide line	f
Narcissus poeticus	ovoid	C	wide line	f
Hippeastrum rutilum	spherical	A	ribbon	a
Hippeastrum vittatum	spherical	A	ribbon	a
