# Supplementary material for: Comparative Plastome Analysis of Three Amaryllidaceae Subfamilies: Insights into Variation of Genome Characteristics, Phylogeny, and Adaptive Evolution
Source: Biomed Res Int. 2022 Mar 24;2022:3909596. doi: 10.1155/2022/3909596 (PMC8970886; doi:10.1155/2022/3909596)
Supplement: Supplementary Materials — Figure S1: comparison of the border regions among the 36 Amaryllidaceae plastid genomes. Figure S2: VISTA-based sequence identity plot of the 36 Amaryllidaceae plastid genomes using Allium fasciculatum as a reference. Figure S3: ML tree based on ITS. Table S1: information and GenBank accessions for sample collection. Table S2: the GenBank accessions of all 41 taxa plastome sequences used this study. Table S3: the GenBank accessions of all 38 taxa ITS sequences used this study. Table S4: number of six SSR types detected in 36 plastid genomes of 36 Amaryllidaceae species. Table S5: number of four repeat types in the plastid genomes of 36 Amaryllidaceae species. Table S6: frequency of four repeat types according to length in 36 Amaryllidaceae species. Table S7: codon usage table contains 14 parameters from 36 plastid genomes of Amaryllidaceae species. Table S8: the 65 protein-coding genes. Table S9: the potential positive selection test based on the branch-site model in Amaryllidoideae. Table S10: the potential positive selection test based on the branch-site model in Agapanthoideae. Table S11: information for two traits of 36 Amaryllidaceae species. [file 3909596.f1.zip › TableS7.pdf]

Table S7 Codon usage table of 5 parameters from 36 plastid genomes of Amaryllidaceae species

**Specie**	CAI	CBI	Fop	ENC	GC3s
Agapanthus coddii	0.17	-0.10	0.35	48.60	0.26
Allium fasciculatum	0.17	-0.11	0.35	47.68	0.25
Allium macranthum	0.17	-0.10	0.35	47.78	0.25
Allium monanthum	0.17	-0.10	0.35	47.75	0.25
Allium fetisowi	0.17	-0.11	0.35	47.39	0.24
Allium neriniflorum	0.17	-0.10	0.35	47.66	0.25
Allium funckiifolium	0.17	-0.11	0.35	47.55	0.25
Allium listera	0.17	-0.11	0.35	47.60	0.25
Allium nanodes	0.17	-0.11	0.35	47.54	0.25
Allium ovalifolium	0.17	-0.11	0.35	47.59	0.25
Allium ovalifolium* var. *cordifolium	0.17	-0.11	0.35	47.56	0.25
Allium ovalifolium* var. *leuconeurum	0.17	-0.11	0.35	47.64	0.25
Allium prattii	0.17	-0.11	0.35	47.50	0.24
Allium victorialis	0.17	-0.11	0.35	47.63	0.25
Allium cyathophorum	0.17	-0.11	0.35	47.36	0.24
Allium mairei	0.17	-0.11	0.35	47.40	0.24
Allium mongolicum	0.17	-0.11	0.35	47.20	0.24
Allium nutans	0.17	-0.11	0.35	47.54	0.25
Allium polyrhizum	0.17	-0.10	0.35	47.23	0.24
Allium przewalskianum	0.17	-0.11	0.35	47.36	0.24
Allium ramosum	0.17	-0.11	0.35	47.60	0.25
Allium tuberosum	0.17	-0.11	0.35	47.83	0.25
Clivia miniata	0.17	-0.09	0.36	49.34	0.27
Hippeastrum rutilum	0.17	-0.10	0.36	49.38	0.27
Hippeastrum vittatum	0.17	-0.09	0.36	49.39	0.27
Leucojum aestivum	0.17	-0.09	0.36	49.07	0.27
Lycoris anhuiensis	0.17	-0.09	0.36	49.05	0.26
Lycoris aurea	0.17	-0.09	0.36	49.02	0.26
Lycoris chinensis	0.17	-0.09	0.36	49.05	0.26
Lycoris longituba	0.17	-0.09	0.36	49.09	0.27
Lycoris radiata	0.17	-0.09	0.36	49.14	0.27
Lycoris sanguinea	0.17	-0.09	0.36	49.09	0.27
Lycoris sprengeri	0.17	-0.09	0.36	49.09	0.27
Lycoris squamigera	0.17	-0.09	0.36	49.02	0.26
Narcissus poeticus	0.17	-0.09	0.36	49.29	0.27
Narcissus tazetta	0.17	-0.09	0.36	49.54	0.27
