# Supplementary material for: Comparative Plastome Analysis of Three Amaryllidaceae Subfamilies: Insights into Variation of Genome Characteristics, Phylogeny, and Adaptive Evolution
Source: Biomed Res Int. 2022 Mar 24;2022:3909596. doi: 10.1155/2022/3909596 (PMC8970886; doi:10.1155/2022/3909596)
Supplement: Supplementary Materials — Figure S1: comparison of the border regions among the 36 Amaryllidaceae plastid genomes. Figure S2: VISTA-based sequence identity plot of the 36 Amaryllidaceae plastid genomes using Allium fasciculatum as a reference. Figure S3: ML tree based on ITS. Table S1: information and GenBank accessions for sample collection. Table S2: the GenBank accessions of all 41 taxa plastome sequences used this study. Table S3: the GenBank accessions of all 38 taxa ITS sequences used this study. Table S4: number of six SSR types detected in 36 plastid genomes of 36 Amaryllidaceae species. Table S5: number of four repeat types in the plastid genomes of 36 Amaryllidaceae species. Table S6: frequency of four repeat types according to length in 36 Amaryllidaceae species. Table S7: codon usage table contains 14 parameters from 36 plastid genomes of Amaryllidaceae species. Table S8: the 65 protein-coding genes. Table S9: the potential positive selection test based on the branch-site model in Amaryllidoideae. Table S10: the potential positive selection test based on the branch-site model in Agapanthoideae. Table S11: information for two traits of 36 Amaryllidaceae species. [file 3909596.f1.zip › TableS8.pdf]

Table S8 65 Protein-coding genes

**Gene name**								
psbA	***ndhA***	***rpl14***	***rps3***	***ccsA***	***petA***	***atpA***	***psaA***	***rpoA***
psbB	***ndhC***	***rpl16***	***rps4***	***clpP***	***petB***	***atpB***	***psaB***	***rpoB***
psbC	***ndhD***	***rpl20***	***rps8***	*matK*	***petD***	***atpE***	***psaC***	***rpoC1***
psbD	***ndhE***	***rpl22***	***rps11***	***rbcL***	***petG***	***atpF***	*psaI*	***rpoC2***
psbE	***ndhF***	*rpl32*	***rps14***	***ycf3***	***petL***	***atpH***	***psaJ***	
psbF	***ndhG***	***rpl33***	***rps16***	***ycf4***	***petN***	***atpI***		
psbH	***ndhH***	***rpl36***	***rps18***	***accD***				
psbI	***ndhI***							
psbJ	***ndhJ***							
psbK	***ndhK***							
psbL								
psbN								
psbT								

The 60 genes marked in bold are used for positive selection analysis
