# Supplementary material for: Comparative Plastome Analysis of Three Amaryllidaceae Subfamilies: Insights into Variation of Genome Characteristics, Phylogeny, and Adaptive Evolution
Source: Biomed Res Int. 2022 Mar 24;2022:3909596. doi: 10.1155/2022/3909596 (PMC8970886; doi:10.1155/2022/3909596)
Supplement: Supplementary Materials — Figure S1: comparison of the border regions among the 36 Amaryllidaceae plastid genomes. Figure S2: VISTA-based sequence identity plot of the 36 Amaryllidaceae plastid genomes using Allium fasciculatum as a reference. Figure S3: ML tree based on ITS. Table S1: information and GenBank accessions for sample collection. Table S2: the GenBank accessions of all 41 taxa plastome sequences used this study. Table S3: the GenBank accessions of all 38 taxa ITS sequences used this study. Table S4: number of six SSR types detected in 36 plastid genomes of 36 Amaryllidaceae species. Table S5: number of four repeat types in the plastid genomes of 36 Amaryllidaceae species. Table S6: frequency of four repeat types according to length in 36 Amaryllidaceae species. Table S7: codon usage table contains 14 parameters from 36 plastid genomes of Amaryllidaceae species. Table S8: the 65 protein-coding genes. Table S9: the potential positive selection test based on the branch-site model in Amaryllidoideae. Table S10: the potential positive selection test based on the branch-site model in Agapanthoideae. Table S11: information for two traits of 36 Amaryllidaceae species. [file 3909596.f1.zip › TableS9 (1).pdf]

Table S9 The potential positive selection test based on the branch-site model in Amaryllidoideae

Gene	Null hypothesis			Alternative hypothesis			Signifificance test	
	lnL	df	omega (w = 1)	lnL	df	omega (w > 1)	BEB	pvalue
ycf3	1124.5	74	1	1124.5	75	1		0.998
psaJ	256.94	74	1	256.94	75	1		1
atpE	899.64	74	1	899.46	75	297.304	51,L,0.732;	0.5456
petL	164.26	74	1	164.26	75	2.81179		1
rpl33	454.52	74	1	454.52	75	1	27,L,0.506;	1
psbA	2040.6	74	1	2040.6	75	1		0.998
petB	1236.4	74	1	1236	75	268.431	139,R,0.809;	0.3507
ndhK	1585.5	74	1	1585.5	75	1		0.9977
petD	969.28	74	1	969.28	75	1		1
atpB	3019.7	74	1	3019.7	75	1	3,I,0.598;	1
rps8	888.41	74	1	888.19	75	75.3286	101,M,0.583;	0.51
rps16	527.45	74	1	527.37	75	24.0103	74,F,0.611;	0.6991
accD	3439.2	74	1	3439.2	75	1		0.9977
psbI	221.02	74	1	221.02	75	1		1
psbJ	229.52	74	1	229.52	75	2.80069		1
ccsA	3188.1	74	1	3188.1	75	1	154,L,0.538;	1
rpl22	1203.2	74	1	1203.2	75	1	118,L,0.566;121,D,0.589;	1
rpl20	922.89	74	1	922.89	75	1		1
psaA	4212.7	74	1	4212.7	75	1		0.9925
rps4	1222.6	74	1	1222.6	75	1		1
rpoC2	10713	74	1	10713	75	1		0.9925
ndhJ	977.4	74	1	977.4	75	1	78,I,0.724;	1
rps18	599.16	74	1	599.16	75	2.74557		1
ndhI	1379.3	74	1	1379.3	75	2.52963	87,R,0.609;	0.9188
rps14	602.48	74	1	602.41	75	6.45762	88,H,0.670;	0.7208
ndhA	3318.1	74	1	3318.1	75	1		1
atpF	1010.7	74	1	1010.7	75	1		1
rbcL	3127.7	74	1	3127.7	75	1		0.9977
atpH	457.92	74	1	457.92	75	2.74576		1
clpP	1136.9	74	1	1136.9	75	1		1
psbN	229.94	74	1	229.94	75	2.90689		1
petG	191.34	74	1	191.33	75	1		0.9211
rpoA	2506.2	74	1	2506.2	75	1		1
ndhE	747.66	74	1	747.66	75	1		1
psbF	193.42	74	1	193.1	75	999	17,I,0.950;	0.4268
psbT	190.01	74	1	190.01	75	2.95637		1
psbH	418.26	74	1	418.26	75	1		0.9836
ndhD	4280.6	74	1	4280.6	75	1	233,S,0.642;252,L,0.594;321,I,0.629;	1
ndhC	655.94	74	1	655.94	75	1		1
ycf4	1157.7	74	1	1157.7	75	2.88361		0.9989
rpl16	1062.2	74	1	1062.2	75	1		1
rpoB	6907.3	74	1	6907.3	75	1		1
petN	142.28	74	1	142.28	75	2.40356		1
psbC	2691.3	74	1	2691.3	75	1		1
psbD	2008.2	74	1	2008.2	75	1		1
psbB	3140	74	1	3140	75	1		1
ndhG	1467	74	1	1467	75	1		1
atpI	415.76	74	1	415.76	75	1		1
petA	1979.5	74	1	1979.5	75	1		1
rps11	957.14	74	1	957.14	75	2.95435		1
ndhH	3117.7	74	1	3117.7	75	1	43,I,0.716;	1
rps3	1693.5	74	1	1693.5	75	1	24,A,0.615;	1
atpA	3270.9	74	1	3271.1	75	2.95606		0.5471
psbE	456.3	74	1	456.3	75	2.60837		1
ndhF	7474.1	74	1	7474.1	75	1		1
psaC	527.13	74	1	527.13	75	1		1
psaB	4158.2	74	1	4158.2	75	1		1
rpl14	822.07	74	1	822.07	75	1		1
rpl36	238.98	74	1	238.98	75	2.95774		1
rpoC1	4660.9	74	1	4660.9	75	1		1

Bold types are genes with positively selected sites. BEB, Bayesian Empirical Bayes.
