# Supplementary figures and images for: Comparative Plastome Analysis of Three Amaryllidaceae Subfamilies: Insights into Variation of Genome Characteristics, Phylogeny, and Adaptive Evolution
Source: Biomed Res Int. 2022 Mar 24;2022:3909596. doi: 10.1155/2022/3909596 (PMC8970886; doi:10.1155/2022/3909596)

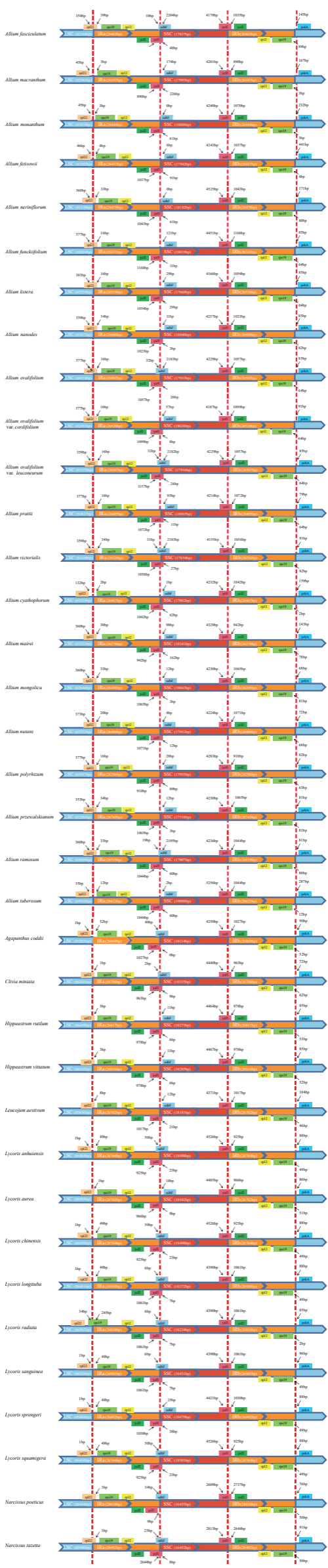

Supplement: Supplementary Materials — Figure S1: comparison of the border regions among the 36 Amaryllidaceae plastid genomes. Figure S2: VISTA-based sequence identity plot of the 36 Amaryllidaceae plastid genomes using Allium fasciculatum as a reference. Figure S3: ML tree based on ITS. Table S1: information and GenBank accessions for sample collection. Table S2: the GenBank accessions of all 41 taxa plastome sequences used this study. Table S3: the GenBank accessions of all 38 taxa ITS sequences used this study. Table S4: number of six SSR types detected in 36 plastid genomes of 36 Amaryllidaceae species. Table S5: number of four repeat types in the plastid genomes of 36 Amaryllidaceae species. Table S6: frequency of four repeat types according to length in 36 Amaryllidaceae species. Table S7: codon usage table contains 14 parameters from 36 plastid genomes of Amaryllidaceae species. Table S8: the 65 protein-coding genes. Table S9: the potential positive selection test based on the branch-site model in Amaryllidoideae. Table S10: the potential positive selection test based on the branch-site model in Agapanthoideae. Table S11: information for two traits of 36 Amaryllidaceae species. [file 3909596.f1.zip › FigureS1 (1).pdf]

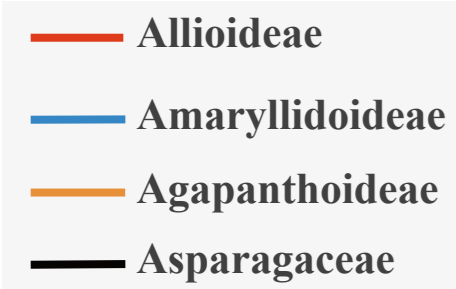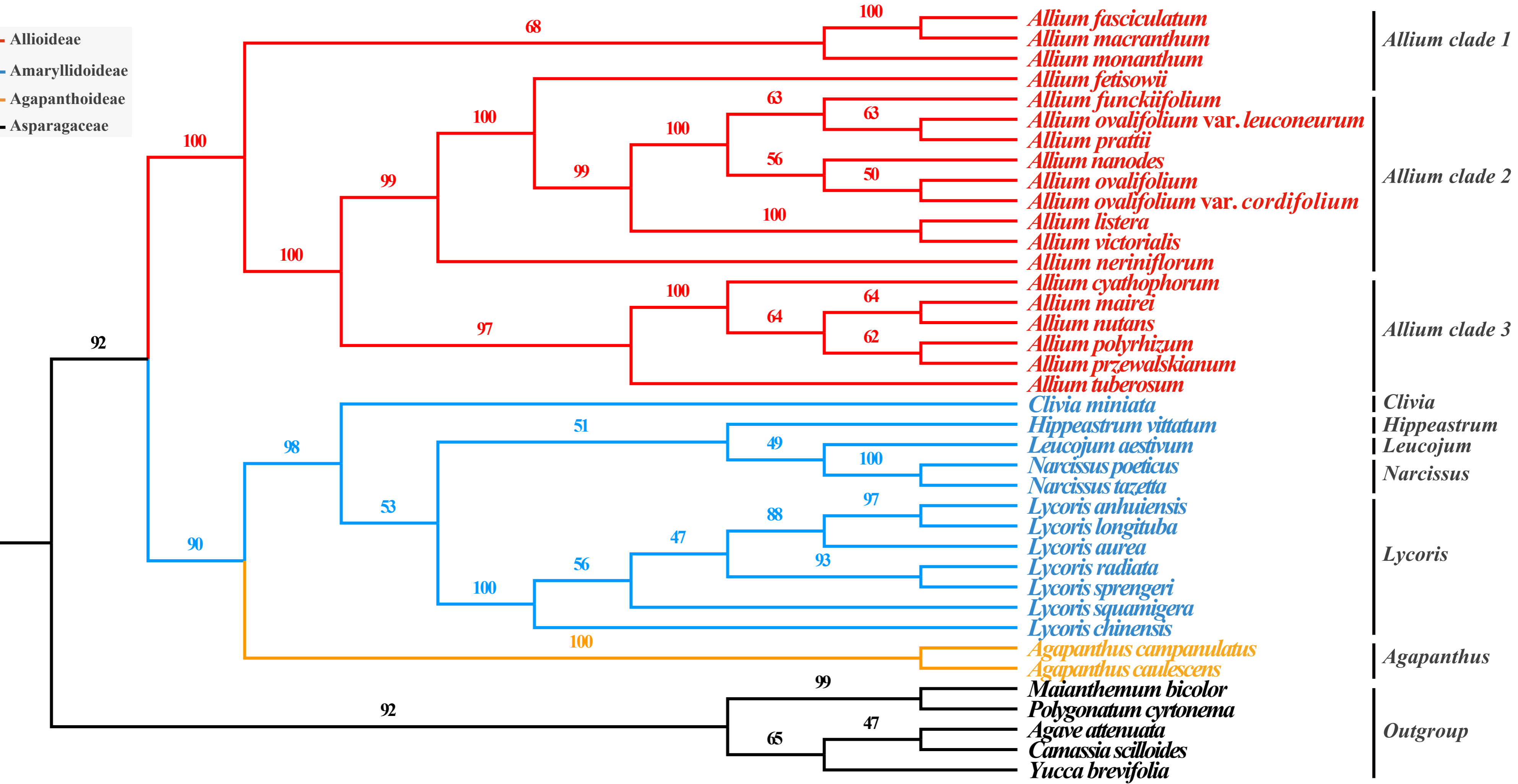

1.1

Supplement: Supplementary Materials — Figure S1: comparison of the border regions among the 36 Amaryllidaceae plastid genomes. Figure S2: VISTA-based sequence identity plot of the 36 Amaryllidaceae plastid genomes using Allium fasciculatum as a reference. Figure S3: ML tree based on ITS. Table S1: information and GenBank accessions for sample collection. Table S2: the GenBank accessions of all 41 taxa plastome sequences used this study. Table S3: the GenBank accessions of all 38 taxa ITS sequences used this study. Table S4: number of six SSR types detected in 36 plastid genomes of 36 Amaryllidaceae species. Table S5: number of four repeat types in the plastid genomes of 36 Amaryllidaceae species. Table S6: frequency of four repeat types according to length in 36 Amaryllidaceae species. Table S7: codon usage table contains 14 parameters from 36 plastid genomes of Amaryllidaceae species. Table S8: the 65 protein-coding genes. Table S9: the potential positive selection test based on the branch-site model in Amaryllidoideae. Table S10: the potential positive selection test based on the branch-site model in Agapanthoideae. Table S11: information for two traits of 36 Amaryllidaceae species. [file 3909596.f1.zip › FigureS3.pdf]
